# Interface Dynamics in Ag–Cu_3_P Nanoparticle Heterostructures

**DOI:** 10.1021/jacs.1c09179

**Published:** 2021-12-24

**Authors:** Michael S. Seifner, Markus Snellman, Ofentse A. Makgae, Krishna Kumar, Daniel Jacobsson, Martin Ek, Knut Deppert, Maria E. Messing, Kimberly A. Dick

**Affiliations:** † Centre for Analysis and Synthesis, Lund University, Box 124, 22100 Lund, Sweden; ‡ NanoLund, Lund University, Box 118, 22100 Lund, Sweden; § Solid State Physics, Lund University, Box 118, 22100 Lund, Sweden; ∥ National Center for High Resolution Electron Microscopy, Lund University, Box 124, 22100 Lund, Sweden

## Abstract

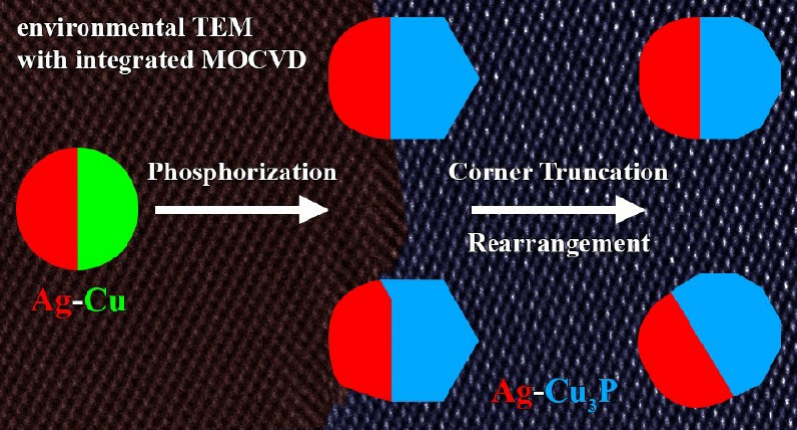

Earth-abundant transition metal phosphides are promising materials for energy-related applications. Specifically, copper(I) phosphide is such a material and shows excellent photocatalytic activity. Currently, there are substantial research efforts to synthesize well-defined metal–semiconductor nanoparticle heterostructures to enhance the photocatalytic performance by an efficient separation of charge carriers. The involved crystal facets and heterointerfaces have a major impact on the efficiency of a heterostructured photocatalyst, which points out the importance of synthesizing potential photocatalysts in a controlled manner and characterizing their structural and morphological properties in detail. In this study, we investigated the interface dynamics occurring around the synthesis of Ag–Cu_3_P nanoparticle heterostructures by a chemical reaction between Ag–Cu nanoparticle heterostructures and phosphine in an environmental transmission electron microscope. The major product of the Cu–Cu_3_P phase transformation using Ag–Cu nanoparticle heterostructures with a defined interface as a template preserved the initially present Ag{111} facet of the heterointerface. After the complete transformation, corner truncation of the faceted Cu_3_P phase led to a physical transformation of the nanoparticle heterostructure. In some cases, the structural rearrangement toward an energetically more favorable heterointerface has been observed and analyzed in detail at the atomic level. The herein-reported results will help better understand dynamic processes in Ag–Cu_3_P nanoparticle heterostructures and enable facet-engineered surface and heterointerface design to tailor their physical properties.

## Introduction

Over the last years, nanostructured transition metal phosphides (TMPs) have been the focus of several research efforts due to their excellent performance in energy-related applications, including electrocatalysis, photocatalysis, and energy storage.^1^ Most of the targeted TMPs with high potential in this research field are earth-abundant and could become a cost-efficient and sustainable alternative compared to widely used noble metals.^2−4^ Copper(I) phosphide (Cu_3_P), which is the focus of this work, is a p-type semiconductor with a bandgap of ∼1.50 eV.^5−7^ The p-type semiconducting behavior of Cu_3_P has its origin in the pronounced substoichiometry in Cu due to Cu vacancies, which has already been discussed in detail in previous studies.^8,9^ A homogeneity range between approximately Cu_2.9_P and Cu_2.3_P has been reported,^10^ making the use of the chemical formula Cu_3–*x*_P more appropriate, and theoretical calculations suggest an increase of Cu vacancies with temperature.^8^ Since we did not study the composition in detail, we will refer to Cu_3–*x*_P as Cu_3_P for simplicity. Cu-deficient Cu_3_P shows a high performance as anode material for Li-ion batteries^10,11^ and even exceeds the volumetric capacity of graphite.^12^ Moreover, the self-doping of Cu_3_P results in efficient and tunable light absorption in the near-infrared region due to localized surface plasmon resonance (LSPR).^5,8,9^ The outstanding properties of Cu_3_P have enabled its application in various fields, including energy storage,^13−17^ photodetection,^18^ catalysis,^19^ sensing,^20,21^ ion exchange,^8,22^ and cancer therapy.^23^ Especially, the use of Cu_3_P as a catalyst for the hydrogen evolution reaction (HER) is a promising and highly investigated research area.^24−33^

Recent studies reveal an increased photocatalytic activity when Cu_3_P is combined with noble metals^34,35^ or n-type semiconductors^6,7,33,36^ due to efficient charge carrier separation by plasmon–exciton coupling^37,38^ or an n-p heterojunction.^39,40^ Various properties within the same nanocrystal, including the facet-dependent photocatalytic activity and the electronic band structure, point out the importance of a facet-engineered surface and heterointerface design.^41−43^ The facet-dependent photocatalytic activity is well-described in the literature and caused by facet-varying reactant adsorption/product desorption as well as charge carrier separation/transfer characteristics.^44−48^ Moreover, the surface termination of a crystal facet can vary with its orientation and process conditions, which has an impact on the photocatalytic activity.^49^ Additionally, the facets forming a heterointerface have been identified as an important factor for the performance of a metal–semiconductor heterostructure as a photocatalyst. This is due to the dependence of the surface work function on the crystal facet and the resulting ability to form the required Schottky junction.^50,51^ The discussed potential of nanoparticle heterostructures is promoted by tremendous research efforts in this field.^52−54^ To the best of our knowledge, the combination of Ag and Cu_3_P with potential application in photocatalysis has not been investigated so far. Therefore, a detailed analysis of such nanoparticle heterostructures’ structural and morphological evolution during the synthesis is required to tailor their properties and correlate them with their photocatalytic performance.

Our study aims to reveal the dynamic processes just before, during, and immediately after synthesizing Ag–Cu_3_P nanoparticle heterostructures by *in situ* transmission electron microscopy (TEM). We performed the experiments by depositing Ag–Cu nanoparticle heterostructures on a heating chip, which we then transferred to an environmental transmission electron microscope (ETEM) with an integrated metal–organic chemical vapor deposition (MOCVD) system. The controlled supply of phosphine (PH_3_) to Ag–Cu nanoparticle heterostructures with defined interfaces allowed for the observation of the Cu–Cu_3_P phase transformation at moderate temperatures. We analyzed the dynamic processes involved in the phase transformation and characterized the present phases *via* high-resolution TEM (HRTEM) imaging, high frame rate TEM movies, and energy dispersive X-ray spectroscopy (EDS). The herein presented Ag–Cu_3_P nanoparticle heterostructures have the potential to act, for instance, as high-performance photocatalysts for water splitting. Our results will help synthesize Ag–Cu_3_P nanoparticle heterostructures with well-defined facets and heterointerfaces *via* a gas-phase process, enabling the evaluation of their impact on the optical properties/potential photocatalytic activity in future studies.

## Results and Discussion

For the formation of Ag–Cu_3_P nanoparticle heterostructures, bimetallic Ag–Cu nanoparticles were generated in a spark ablation system,^55^ and particles with a defined diameter of 30 nm were selected *via* an integrated filtering system for the deposition on a microelectromechanical systems (MEMS)-based heating chip for *in situ* TEM investigations. Subsequently, the chip was transferred to an ETEM with an integrated MOCVD system. The deposited bimetallic nanoparticles were heated to 350 °C and revealed phase separation with rough heterointerfaces and the presence of surface oxides/C-based contamination (see Figure S1). Therefore, the initially present oxides were reduced *via* a H_2_ treatment at 500–650 °C, resulting in the complete removal of surface oxides and the formation of sharp heterointerfaces. The observation of a large number of Ag–Cu nanoparticle heterostructures with a sharp Ag{111}/Cu{111} interface (see Figure S2) is in good agreement with a previous experimental study performed under comparable conditions, in which the side-by-side orientation of the Ag and Cu phases connected *via* {111} facets was identified as the energetically most favorable structural configuration for the here applied size range.^56^ However, for a minority of particles, twinned phases and rough heterointerfaces remained even after the H_2_ annealing (see Figure S3).

Subsequently, the temperature was reduced to 350 °C, and PH_3_ was supplied to the system to initiate the Cu–Cu_3_P phase transformation. The analysis of heterointerfaces in the resulting Ag–Cu_3_P nanoparticles suggests that the major product after the phase transformation had a Ag{111}/Cu_3_P{3300} interface (see Figure S4). The preserved Ag{111} facet, in combination with the in-plane angular relation between the Ag and Cu phases as well as the Cu and Cu_3_P phases (explained in detail below), suggests the initially present Ag{111}/Cu{111} interface acting as a template. Nevertheless, a minor fraction of nanoparticle heterostructures with different facets forming the Ag–Cu_3_P interface has been observed, which indicates scenarios where the Ag{111}/Cu_3_P{3300} interface cannot be maintained.

**Figure 1:**
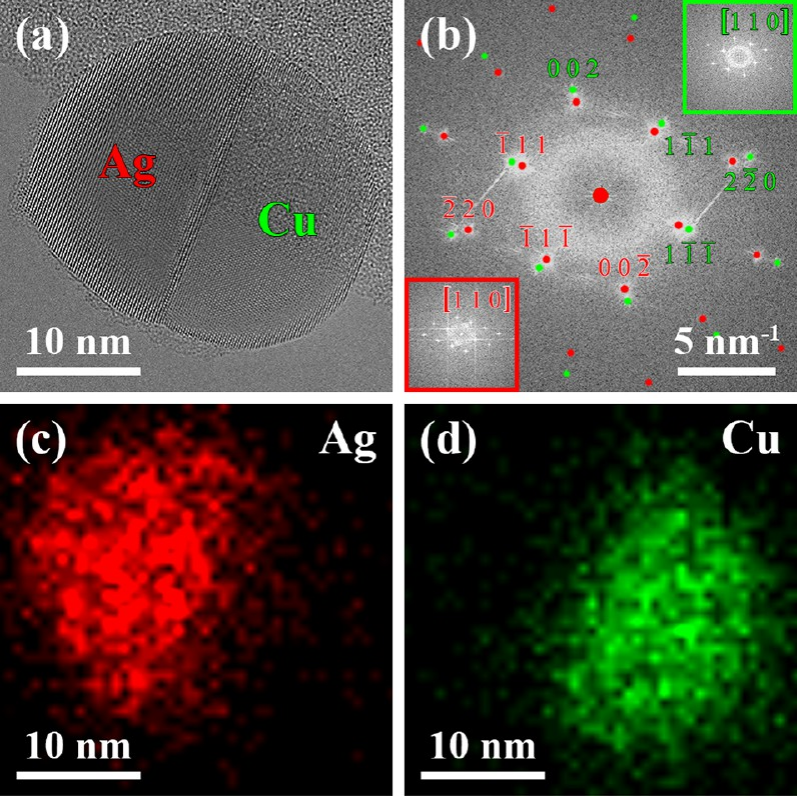
(a) HRTEM image of an Ag–Cu nanoparticle heterostructure acquired at 350 °C after H_2_ treatment at 500 °C. (b) Corresponding power spectrum with overlaid simulated electron diffraction patterns^57^ of the cubic Ag (red) and Cu (green) phases (space group: *Fm*3*m*). The power spectra of Ag (red, bottom left) and Cu (green, top right) as insets in (b) highlight the successful tilting of both phases close to their [110] zone axes. Consequently, the heterointerface in (a) is near-parallel to the direction of the electron beam. (c) Ag (Lα_1_) and (d) Cu (Kα_1_) STEM-EDS elemental maps of the Ag–Cu nanoparticle heterostructure shown in (a).

At this point, it was unclear if uncommon structural configurations of Ag–Cu_3_P nanoparticle heterostructures are formed solely by Ag–Cu nanoparticles without a sharp Ag{111}/Cu{111} interface (see Figure S3) or if there is an alternative pathway for Ag–Cu nanoparticles with a sharp Ag{111}/Cu{111} interface. Therefore, a Ag–Cu nanoparticle heterostructure was tilted until the interface was oriented near-parallel to the electron beam and both phases oriented close to one of their zone axes (Figure 1). The power spectra shown as insets in Figure 1b correspond to the cubic Ag (red) and Cu (green) phases and confirm the successful tilting close to their [110] zone axes. The acquired HRTEM image (Figure 1a) and the corresponding power spectrum with overlaid simulated electron diffraction patterns^57^ of the Ag and Cu phases with space group *Fm*3*m* (Figure 1b) show their heterointerfacial connection *via* {111} facets. Note that the appearance of lattice fringes is not sensitive to small tilts up to a few degrees of the crystal away from the intended zone axes, given the imaging parameters and low thicknesses. Hence, we qualify their orientations as being close to [110]. However, note that, even with a few degrees of crystal tilt as observed for the nanoparticle heterostructure in Figure 1b, it is correct to say that the interface consists primarily of connected {111} planes, with the potential presence of a few steps to account for relative crystal tilts.^56^ This analysis also applies to later examples of (near) parallel lattice planes at interfaces.

Moreover, scanning transmission electron microscopy (STEM)-EDS elemental maps acquired at 350 °C (Figure 1c,d) confirm the given attribution of the Ag and Cu phases. The selected area sum EDS spectra reveal the high purity of both phases noticeable by a low solubility of less than 3 atom % of Ag in Cu and *vice versa* (see Figure S5), which is in accordance with the Ag–Cu binary phase diagram.^59^

**Figure 2:**
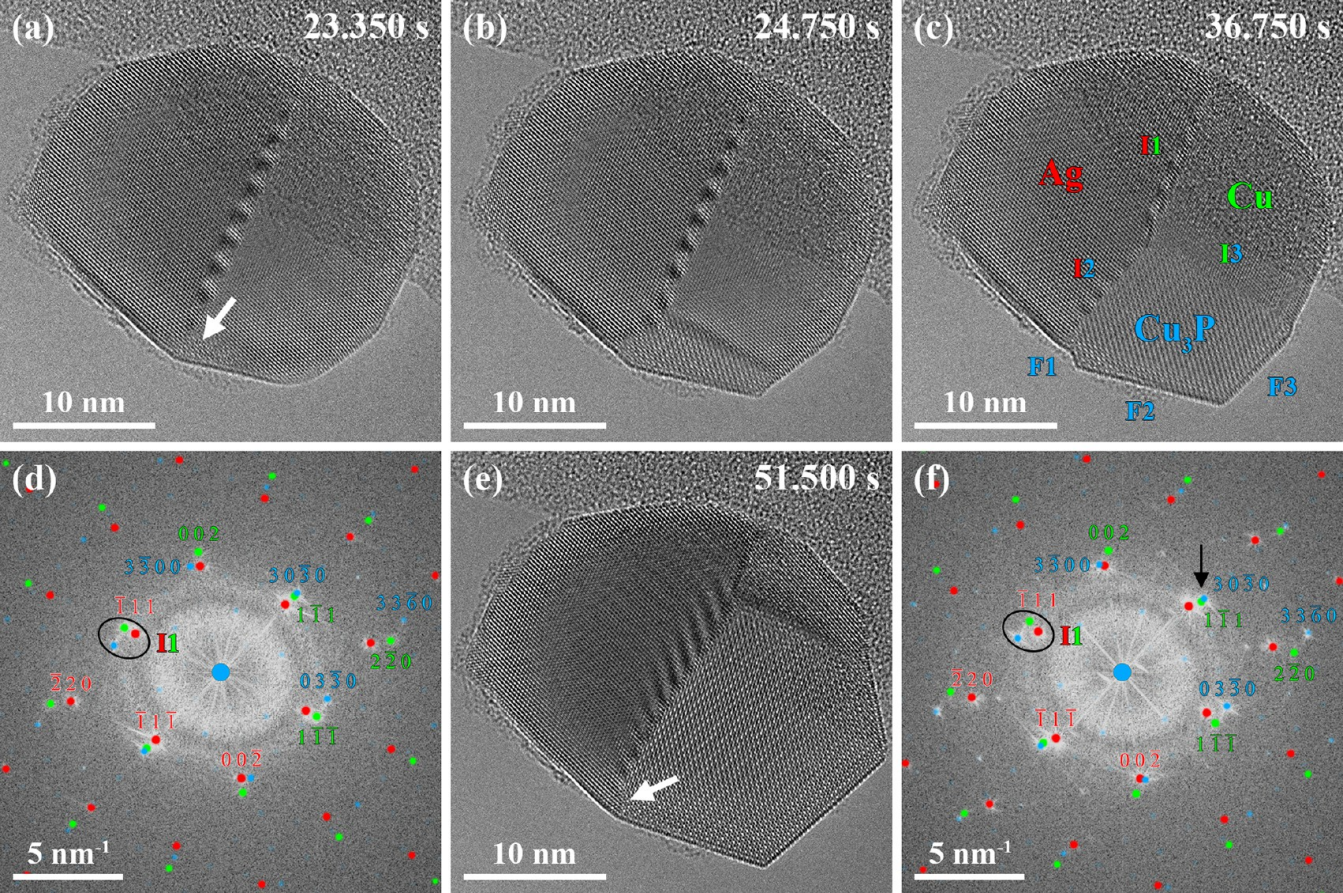
Selected averaged frames of a HRTEM movie (see Movie S1) at (a) 23.350 s, (b) 24.750 s, (c) 36.750 s, and (e) 51.500 s after the supply of PH_3_ to the Ag–Cu nanoparticle heterostructure shown in Figure 1, which was kept at 350 °C. (a) Cu_3_P phase nucleates at the triple-phase boundary indicated by a white arrow and (b) grows by consuming the Cu phase. (c) Phase transformation is far advanced, and the different phases, the heterointerfaces I1–3, and the Cu_3_P facets F1–3 are highlighted. (d) Power spectrum corresponding to (c) with overlaid simulated electron diffraction patterns of the different phases^57,58^ shows their crystallographic relation, and the black ellipse indicates the Ag(111)/Cu(111) heterointerface I1, which remains unaltered when compared to the power spectrum in Figure 1b. The Cu_3_P phase (blue) is oriented close to its [0001] zone axis. (e) Strong fringes at the heterointerface I2 caused by strain effects are visible just before the complete transformation of the Cu phase. The white arrow highlights the wetting of the Cu_3_P facet F1 by Ag as part of the structural rearrangement. (f) Simulated reflections in the black ellipse match with the power spectrum corresponding to (e) and show an anticlockwise in-plane rotation of the Ag phase relative to the Cu and Cu_3_P phases when compared with those in (d). The Cu(111) planes show an in-plane angular relation with Cu_3_P(3030) planes during the rearrangement, which is indicated by the black arrow in (f) and also observable in (d).

As part of the phase transformation experiment, an HRTEM movie (see Movie S1) of the same Ag–Cu nanoparticle heterostructure as highlighted in Figure 1 was acquired to capture the dynamic processes occurring straight after the supply of PH_3_. A sequence of selected averaged frames of this movie reveals a phase transformation of the Cu phase (Figure 2). The phase transformation started 23.350 s after the supply of PH_3_ at the triple-phase boundary of the Ag–Cu nanoparticle heterostructure. A white arrow in Figure 2a indicates the location of the nucleation event of the new phase. The formed phase (Figure 2a–c) can be assigned to hexagonal Cu_3_P (blue, space group: *P*6_3_*cm*) oriented close to its [0001] zone axis *via* analysis of the power spectrum (Figure 2d) corresponding to the HRTEM image in Figure 2c. The formation of Cu_3_P proceeded by consuming Cu at the Cu–Cu_3_P interface (I3); however, a detailed analysis of the local atomic structure of this specific heterointerface is not possible due to the fast dynamic processes caused by the ongoing chemical reaction.

The Ag(111)/Cu(111) interface (I1) dominated the arrangement of the three phases in Figure 2c, which is indicated by the unaltered in-plane angular alignment of the corresponding Ag and Cu reflections in the black ellipse in Figure 2d when compared to Figure 1b. The formation of Cu_3_P{3360} facets, which are highlighted as facets F1–3 in Figure 2c, are attributed to the Cu–Cu_3_P phase transformation. This observation is consistent with previously reported faceting of Cu_3_P nanoparticles with a similar size obtained by a solution-based approach including surface-active agents.^34^ This leads to the conclusion that the different process conditions, including the presence of the Ag phase, the surrounding gas phase, and the low pressure in the ETEM, did not alter the facet evolution of the Cu_3_P phase. It should be noted that facets F1–3 might also be formed by two different facets resulting in an edge, which is illustrated elsewhere.^60^

Moreover, the growth of the Cu_3_P phase was accompanied by a volume expansion of ∼43% (related to the Cu phase) and caused a massive strain in the heterostructure (Figure 2e), resulting in fringes located in the Ag phase close to the Ag–Cu_3_P interface (I2). Upon further progress of the phase transformation, the area of heterointerface I1 decreased (Figure 2e). A comparison of the corresponding reflections in the black ellipses in Figure 2d,f suggests the occurrence of an anticlockwise in-plane rotation of the Ag phase relative to the Cu and Cu_3_P phases about the associated zone axis. It is worth mentioning that the Cu(111) planes showed an in-plane angular relation with the Cu_3_P(3030) planes during this rearrangement, indicated by the black arrow in Figure 2f and in agreement with a previous study on the Cu–Cu_3_P phase transformation.^60^ The increasing impact of the in-plane angular mismatch between the Ag and Cu_3_P phases and the simultaneously decreasing area of heterointerface I1 might have been the driving force for the anticlockwise in-plane rotation of the Ag phase relative to the Cu and Cu_3_P phases. Moreover, the relative in-plane rotation led to (or might have been initiated by) Ag wetting the facet F1, which is highlighted by a white arrow in Figure 2e.

**Figure 3:**
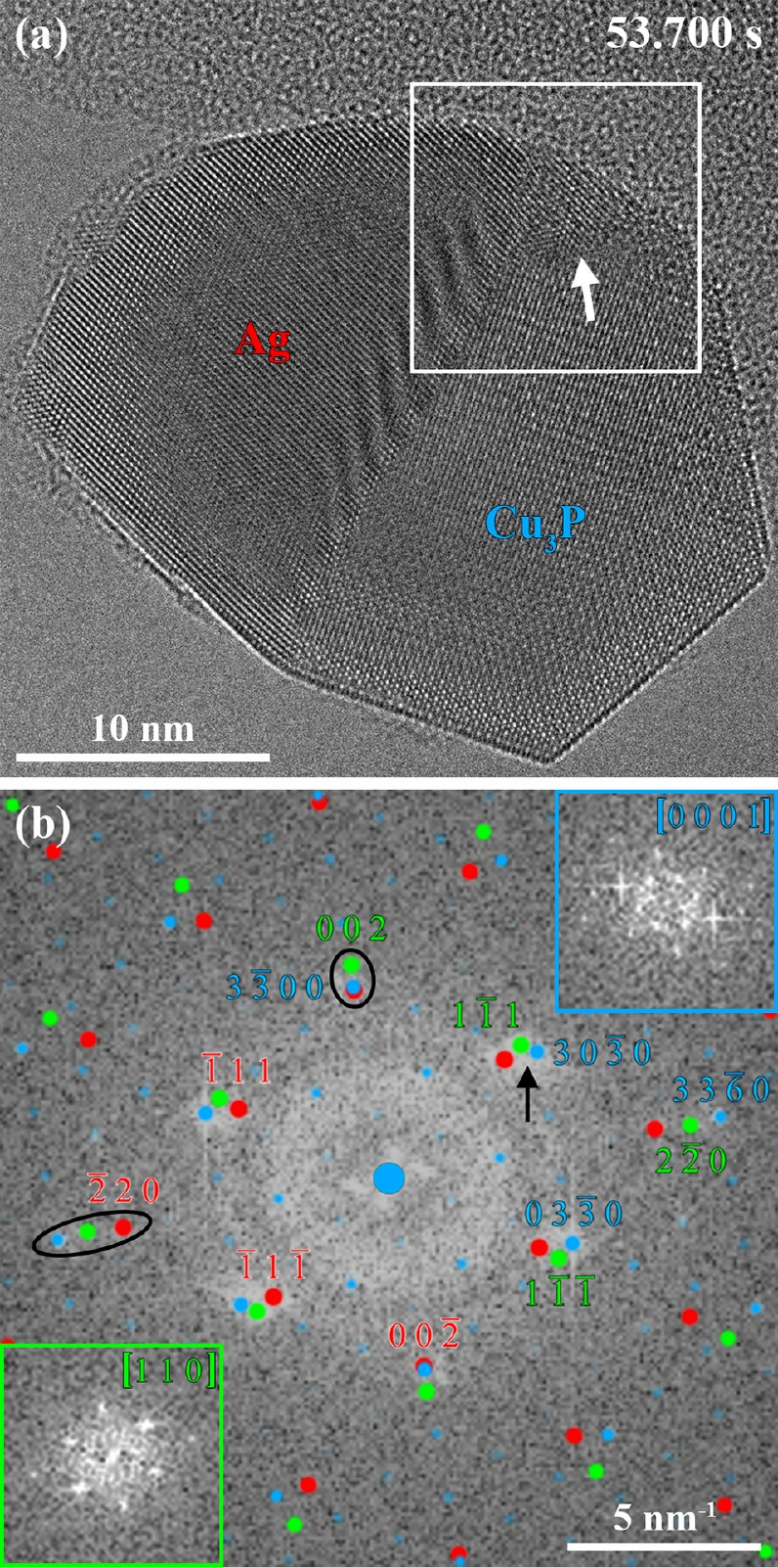
(a) Selected averaged frames of the same HRTEM movie used in Figure 2 (see Movie S1). The reaction between Cu and PH_3_ is nearly completed 53.700 s after the supply of PH_3_, and a white arrow indicates the location of the Cu phase. The power spectrum in (b) corresponds to the white rectangle in (a), and the overlaid simulated electron diffraction patterns of the three involved phases show that the in-plane angular relation between the Cu(111) and Cu_3_P(3030) planes is slightly altered (black arrow). Instead, the Ag, Cu, and Cu_3_P reflections highlighted *via* black ellipses in (b) reveal a well-matching in-plane angular alignment. The power spectra of Cu (green, bottom left) and Cu_3_P (blue, top right) are shown as insets in (b).

A white arrow in Figure 3a indicates the remaining Cu phase 53.700 s after the supply of PH_3_. The power spectrum obtained from the region highlighted by a white rectangle in Figure 3a and overlaid with simulated electron diffraction patterns from the involved phases reveals an alteration of the crystallographic arrangement of the phases in the last stage of the phase transformation (Figure 3b). The in-plane angular relation between the Cu(111) and Cu_3_P(3030) planes was slightly altered compared to Figure 2d,f. Instead, the phases were arranged so that the Ag, Cu, and Cu_3_P reflections located within the black ellipses in Figure 3b have the same in-plane angular alignment. We believe that the alteration of the addressed in-plane angular relation between Cu and Cu_3_P is the key step to form Ag–Cu_3_P nanoparticle heterostructures with uncommon structural configurations discussed below.

**Figure 4:**
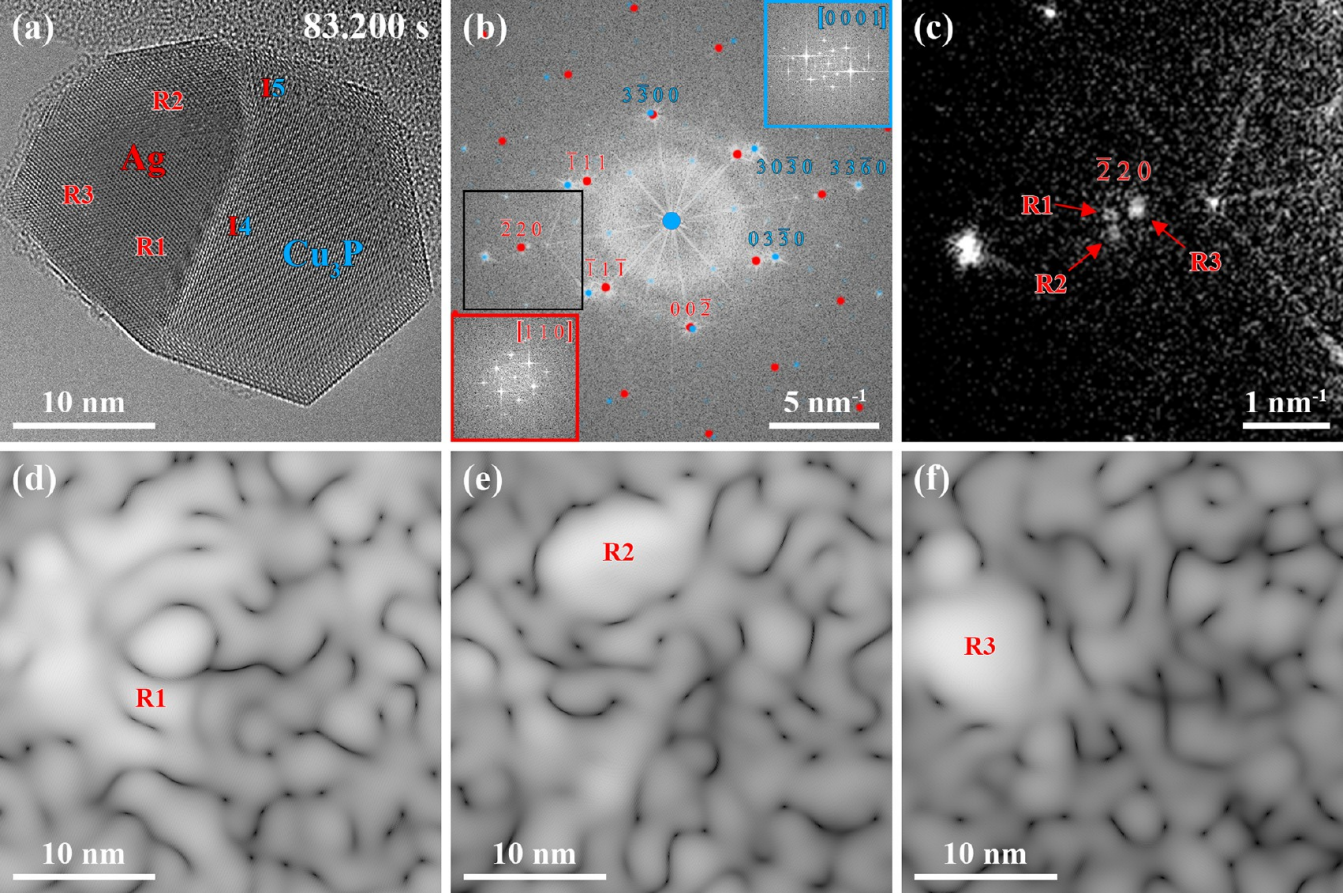
(a) Selected averaged frames of an HRTEM movie (see Movie S1) representing the state of the Ag–Cu_3_P nanoparticle heterostructure 83.200 s after the supply of PH_3_. The phase transformation is completed, and two heterointerfaces, I4 and I5, are visible. Moreover, fringes are located in the Ag phase close to heterointerface I4, which indicates strain effects. (b) Power spectrum corresponding to (a) with overlaid simulated Ag and Cu_3_P electron diffraction patterns. The insets in (b) represent power spectra from the Ag (red, bottom left) and the Cu_3_P (blue, top right) phases. (c) Zoomed-in section of the power spectrum (brightness and contrast adapted) indicated by a black rectangle in (b) shows the Ag(220) reflections. Three slightly different Ag(220) reflections labeled as regions R1–3 are highlighted by red arrows. (d–f) Inversed power spectra after applying a spot mask on (d) R1, (e) R2, and (f) R3 and their associated Ag(220) reflections in the power spectrum in (b). The reflections in (c) can be assigned to different regions within the Ag phase, highlighted in (a).

In addition to the Ag(111)/Cu_3_P(0330) interface (I4), a new Ag(220)/Cu_3_P(3360) interface (I5) evolved after Cu had reacted completely with PH_3_ to form Cu_3_P (Figure 4a). Several incidents during the phase transformation, including the significant volume expansion during the Cu–Cu_3_P phase transformation, the structural rearrangement of the phases just before the complete transformation (Figure 3), and the strained Ag phase at heterointerface I4, have been observed and might have contributed to the formation of heterointerface I5. However, we identified the presence of a defect in the Cu phase (see Figure S6) located in the region where heterointerface I5 started to grow, as the major difference to other Ag–Cu nanoparticle heterostructures and therefore as the potential origin of this observation. The assignment of heterointerfaces to the aforementioned planes does not strictly require that they are parallel. Minimal relative out-of-plane rotations cannot be ruled out on the basis of the appearance of the corresponding lattice fringes. However, these can be accommodated through steps or dislocations without changing the plane-types constituting the majority of the heterointerface. Analysis of the contrast in HRTEM images, detailed for the images acquired after the completion of the reaction, shows that any such out-of-plane rotations are minimal.

The appearance of heterointerface I5 prevented a free rotation of the phases, which is why fringes expanding over the Ag phase close to heterointerface I4 were still present after the consumption of Cu. In Ag–Cu_3_P nanoparticle heterostructures with a single Ag(111)/Cu_3_P(0330) interface—and therefore the freedom of the phases to rotate—such fringes are not visible (see Figure S4). The power spectrum of the HRTEM image in Figure 4a confirms the complete consumption of the Cu phase, and the insets reveal that the Ag and the Cu_3_P phases were still oriented close to their same zone axes (Figure 4b).

A closer look at the Ag(220) reflections points out slight variations of corresponding planes in their in-plane orientation and/or interplanar spacing (Figure 4c). A spot mask on each of those three reflections has been applied, and subsequently, the obtained power spectra have been inversed (Figure 4d–f). Each set of reflections can be assigned to different regions R1–3 within the Ag phase. Region R1 is located close to the surface of the Ag phase and the area where Ag forms heterointerface I4. Region R2 can be associated with the Ag phase forming heterointerface I5, while region R3 is not in contact with the Cu_3_P phase. Ag(220) planes in regions R1 and R2 have similar interplanar spacings; however, an in-plane angular misfit exists. In contrast, Ag(220) planes in regions R1 and R3 do not show an in-plane angular misfit, but the interplanar spacing is larger in region R3. The different regions in the Ag phase, also highlighted in Figure 4a, suggest an inhomogeneous strain distribution.

**Figure 5:**
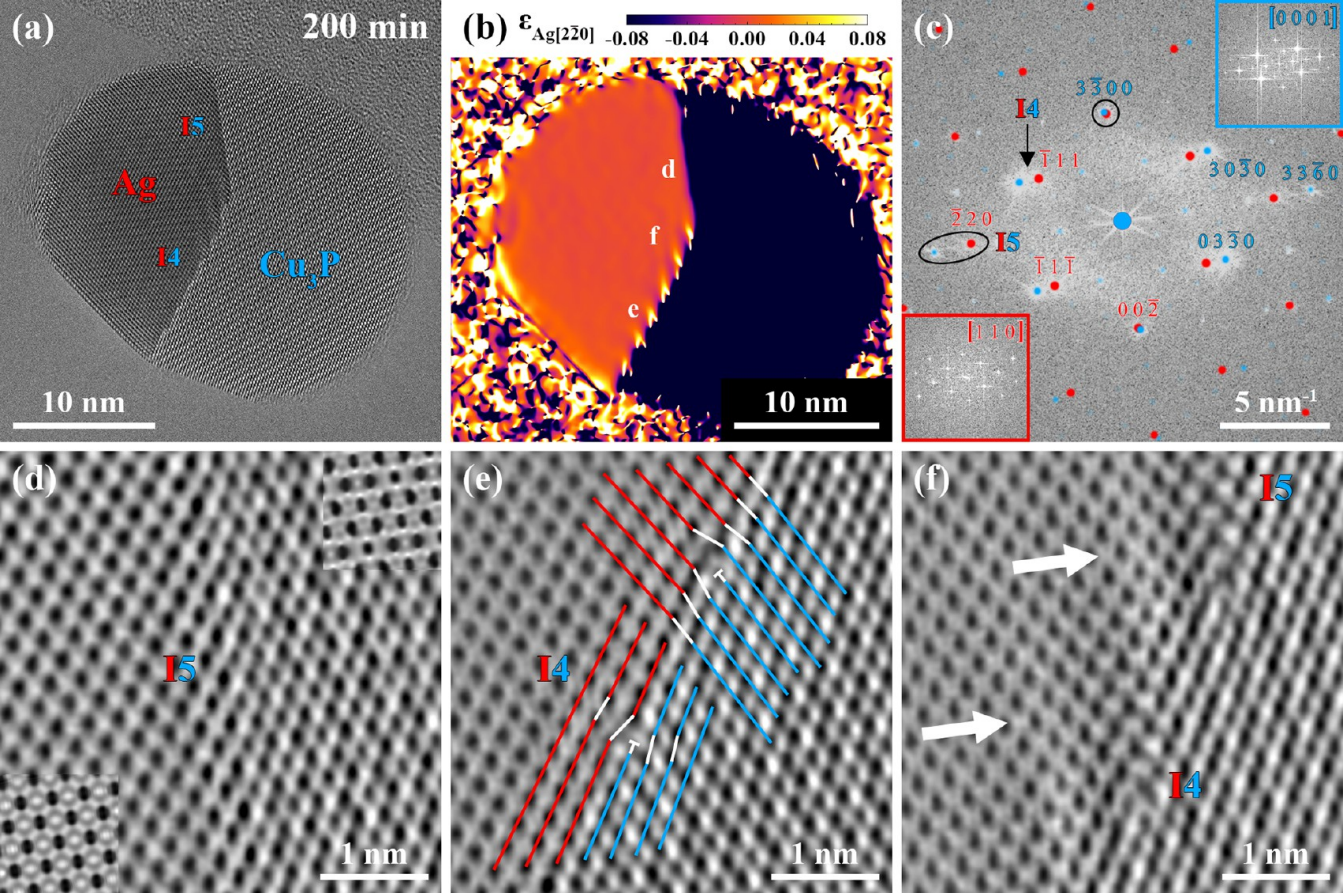
(a) HRTEM image of the same Ag–Cu_3_P nanoparticle heterostructure acquired 200 min after the start of the PH_3_ supply. The temperature and the PH_3_ flow remained unaltered during this period, and heterointerface I5 grew at the cost of heterointerface I4. (b) Strain map of the Ag–Cu_3_P nanoparticle heterostructure obtained from GPA of the phase image of the reconstructed exit wave function. The spot masks for the GPA were centered at the Ag(111) and Ag(111) reflections and the map visualizes strain in the Ag[220]/Cu_3_P[3360] direction. The labels highlight the observed regions in (d–f). (c) Power spectrum corresponding to (a) with overlaid simulated electron diffraction patterns of the Ag and Cu_3_P phases allows for the assignment of well-aligned Ag(220) and Cu_3_P(3360) planes forming heterointerface I5 (black ellipse). The matching Ag (red) and Cu_3_P (blue) reflections in the black circle indicate that the planes perpendicular to heterointerface I5 have the same interplanar spacing in both phases, which could hint toward the origin of the rearrangement to a more stable configuration. The power spectra of the Ag (red, bottom left) and Cu_3_P (blue, top right) phases are shown as insets of (c). (d) Phase image of heterointerface I5 with simulated phase images of the Ag (bottom left) and Cu_3_P (top right) phases as insets. (e) Phase image of the stepped heterointerface I4. Red (Ag) and blue (Cu_3_P) lines indicate the presence of dislocations due to lattice misfit. Ag(111) and Cu_3_P(0330) planes straight at the heterointerface have a parallel in-plane alignment while showing an in-plane rotation further away from the heterointerface. (f) Phase image with fringes (indicated by white arrows) due to strain effects evolving in the Ag phase parallel to heterointerface I5.

Subsequently, after the phase transformation, the nanoparticle heterostructure was kept at 350 °C for a longer period to investigate dynamic processes, including potential interface rearrangement. The HRTEM image in Figure 5a shows the Ag–Cu_3_P nanoparticle heterostructure 200 min after the PH_3_ supply was started. The characterization *via* STEM-EDS confirms the exclusive chemical reaction of PH_3_ with Cu under the chosen process conditions (see Figure S7), which is in good agreement with the ternary phase diagram.^61^ Under the used conditions, the nanoparticle heterostructure underwent a structural rearrangement process, involving the growth of heterointerface I5 at the cost of heterointerface I4 (Figure 5a). Furthermore, corner truncation of the faceted Cu_3_P phase has been observed over time, which is a well-described phenomenon reported for faceted nanoparticles upon thermal annealing and driven by the minimization of the total surface free energy.^62^ We believe that this corner truncation is not related to the presence of multiple heterointerfaces since it was also observed for an Ag–Cu_3_P nanoparticle heterostructure with a single heterointerface (see Figure S8).

A structural evaluation of the heterointerfaces I4 and I5 at an atomic level is possible *via* the reconstruction of the specimen exit wave function from a series of HRTEM images acquired at different defoci.^63^ The phase image of the reconstructed exit wave function was used to prepare a strain map of the Ag–Cu_3_P nanoparticle heterostructure. The spot masks for the geometrical phase analysis (GPA)^64^ were centered at the Ag(111) and Ag(111) reflections. Due to the close proximity of the Cu_3_P(0330) and Cu_3_P(3030) reflections, the Cu_3_P phase was also included in the strain map. The strain across the nanoparticle heterostructure in the Ag[220] and corresponding Cu_3_P[3360] direction is visualized in Figure 5b and shows the different interplanar spacings of the different phases.

The strain map highlights the current conditions at and around the heterointerfaces. For heterointerface I5, a sharp transition from one phase to the other is observed. The well-matching Ag and Cu_3_P reflections in the black ellipse in the power spectrum corresponding to Figure 5a (Figure 5c) suggest that parallel Ag(220) and Cu_3_P(3360) planes form heterointerface I5 (Figure 5d). A minimal out-of-plane rotation of the two phases relative to each other cannot be ruled out based solely on the appearance of the described reflections. However, through comparisons of experimental data and simulations (HRTEM and selected area electron diffraction (SAED)), crystal tilts of both phases off the specific zone axes are determined to have the same direction and the same magnitude of less than 1.6° (see Figures S9–S14). The combination of those results (axis of the rotation is normal to heterointerface I5) and the lack of Moiré fringes observed at heterointerface I5 allows for the exclusion of any out-of-plane rotations. Additionally, heterointerface I5 completely lacks the strain fields observed at heterointerface I4 due to its low-angle mismatch (see below). This supports the assignment of a parallel Ag(220)/Cu_3_P(3360) interface for heterointerface I5.

Moreover, the Ag(002) and Cu_3_P(3300) reflections overlap, highlighted by the black circle in Figure 5c. Consequently, the associated planes (Ag(002) and Cu_3_P(3300)) perpendicular to heterointerface I5 have the same interplanar spacings. This may explain why heterointerface I5 is energetically more favorable than heterointerface I4, which is a kinetic product and formed due to the initially present Ag(111)/Cu(111) interface acting as a template.

In contrast, the combination of a strain map (Figure 5b) and HRTEM image (Figure 5a) reveals a stepped heterointerface I4 with fringes evolving into the Ag phase. The black arrow in the power spectrum corresponding to Figure 5a highlights the in-plane angular misfit of the Ag(111) and Cu_3_P(0330) planes being involved in the formation of heterointerface I4 (Figure 5c). The phase image in Figure 5e reveals a series of misfit dislocations, highlighted by white symbols (T) in Figure 5e, causing the stepped appearance of heterointerface I4. In the here presented case, the dislocations are arranged so that, at each indicated position, both types of differently oriented dislocation lines share the same core (only one type per position indicated).

The complex arrangement of misfit dislocations allows for the accommodation of strain caused by lattice mismatch. Accommodation of strain *via* the formation of dislocations is a well-known mechanism in nanoscale heterostructures.^65,66^ Moreover, the addressed arrangement of line defects enables a more parallel alignment of planes straight at heterointerface I4 to overcome the in-plane angular misalignment of Ag(111) and Cu_3_P(0330) planes further away from the heterointerface. This is indicated by the red (Ag) and blue (Cu_3_P) lines in Figure 5e. Additionally, the defective heterointerface I4 leads to strain in the Ag phase along the Ag[220] direction, which is indicated by white arrows in the phase image in Figure 5f. Besides the in-plane angular misalignment of planes forming heterointerface I4, a comparison of heterointerface I4 (Figure 5) with other nanoparticle heterostructures exhibiting just a single heterointerface formed by the same Ag and Cu_3_P planes (see Figure S4) hints toward a slight rotation of heterointerface I4’s phases against each other, away from the favorable configuration (see Figure S15).

**Figure 6:**
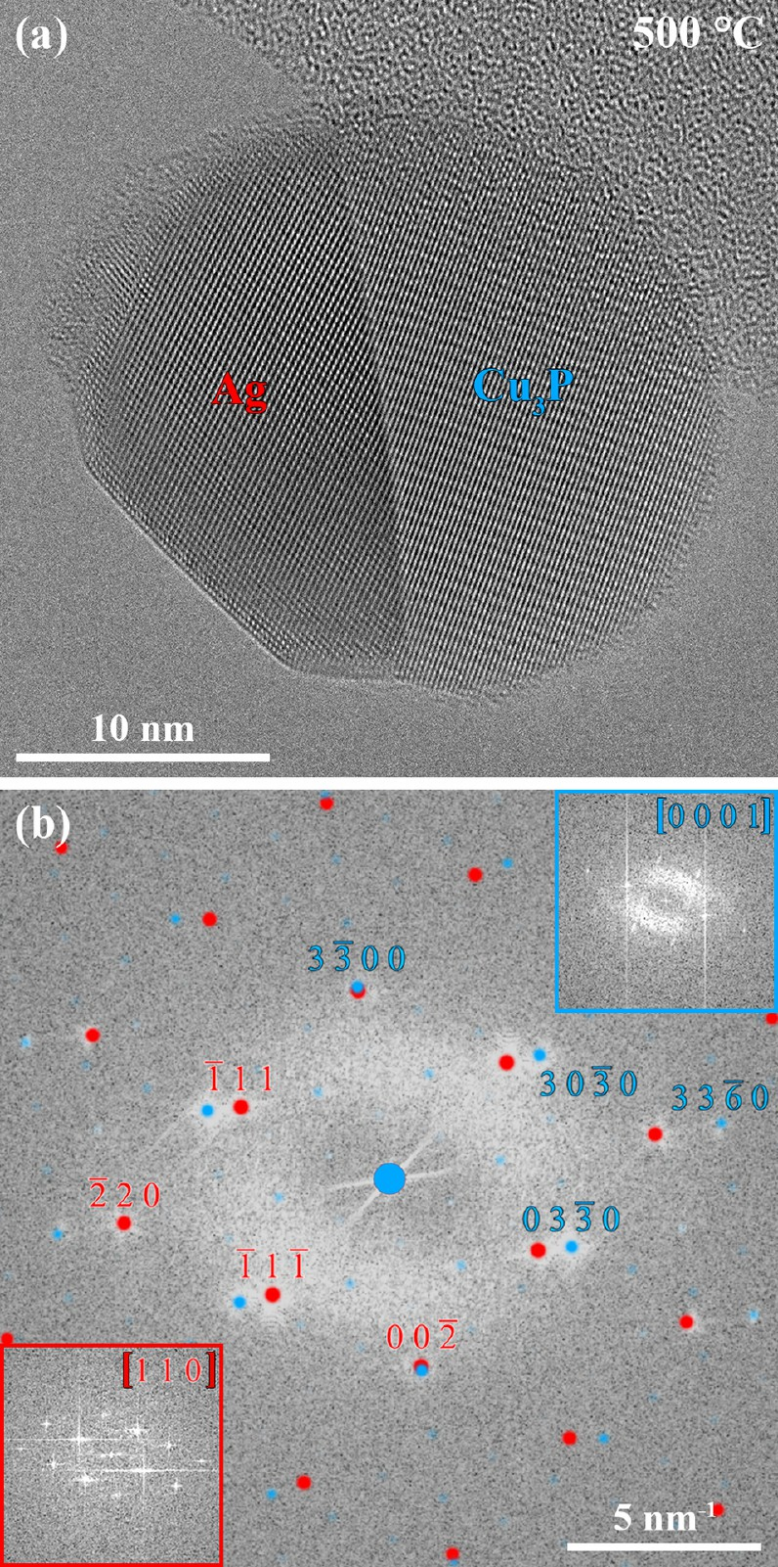
(a) HRTEM image of the same Ag–Cu_3_P nanoparticle heterostructure after increasing the temperature to 500 °C shows the complete replacement of heterointerface I4 by heterointerface I5. (b) Power spectrum corresponding to (a) with overlaid simulated electron diffraction patterns of the Ag and Cu_3_P phases. The thermal expansion of the Ag phase upon heating the sample to 500 °C has been considered for the simulation. The insets in (b) reveal selected area power spectra of the Ag (red, bottom left) and Cu_3_P (blue, top right) phases.

The incoherent heterointerface I4 enabled the efficient diffusion of atoms toward heterointerface I5 due to its high mobility. However, the structural rearrangement of the phases at 350 °C was not finished 217 min after starting the PH_3_ supply. Therefore, the temperature was increased to 500 °C with a heating rate of 5 °C/s to accelerate the rearrangement, and the dynamic processes were tracked by acquiring a HRTEM movie. Heterointerface I5 has almost completely replaced heterointerface I4 during the heating (see Movie S2). After reaching 500 °C, the Ag–Cu_3_P nanoparticle heterostructure reached its stable configuration and heterointerface I4 disappeared, as shown in the HRTEM image in Figure 6a. The final structure did not show fringes expanding from the heterointerface, which hints toward a fully coherent heterointerface. The corresponding power spectrum in Figure 6b only shows minor alterations (the orientation of the nanoparticle heterostructure changed slightly) when compared to the power spectrum of the Ag–Cu_3_P nanoparticle heterostructure before the heat treatment at 500 °C (Figure 5c).

It is also worth mentioning that the presence of C-based contamination (mainly on the Ag surface) was observed during the experiment. This is a well-known observation in electron microscopy and caused by the electron beam in combination with the presence of organic molecules.^67^ Surface contamination could have an impact on the evolution of the Ag facets and, therefore, could contribute to structural rearrangement processes. Furthermore, the energy input by electrons from the electron beam could accelerate the dynamic processes reported in this study. Nevertheless, our study points out the potential of facet-engineered surface and heterointerface design in Ag–Cu_3_P nanoparticle heterostructures by controlling the structural evolution of Ag–Cu nanoparticle templates (e.g., by adapting the particle size^56^) and/or thermal annealing.

## Conclusions

Our results enlighten the dynamic processes that occurred in Ag–Cu nanoparticle heterostructures with a Ag{111}/Cu{111} interface during PH_3_ exposure at 350 °C. The Ag phase did not react with PH_3_ under the used process conditions, while the Cu phase showed a phase transformation into Cu_3_P. Ag–Cu_3_P nanoparticle heterostructures with a single Ag{111}/Cu_3_P{3300} interface were the most common product of this synthesis procedure due to Ag–Cu nanoparticle heterostructures with Ag{111} and Cu{111} facets forming a defined interface acting as a template.

However, in a specific case, we observed dynamic processes leading to the structural rearrangement of the nanoparticle heterostructure. At the beginning of the phase transformation, the arrangement of the three-phase nanoparticle was dominated by the Ag(111)/Cu(111) interface. At a later stage of the transformation, when the Ag/Cu interface area decreased, we observed an in-plane rotation of the Ag phase relative to the Cu and Cu_3_P phases that reduced the in-plane angular misfit between the Ag and Cu_3_P phases. Further, the Cu(111) and Cu_3_P(3030) planes showed an in-plane angular relation during the transformation. In the last steps of the Cu–Cu_3_P phase transformation, when the Cu phase was almost completely consumed, the in-plane angular relation between the Cu(111) and Cu_3_P(3030) planes was slightly altered and an additional heterointerface—formed by Ag(220) and Cu_3_P(3360) planes—appeared. We believe that the formation of the Ag(220)/Cu_3_P(3360) interface was caused by an initially present defect in the Cu phase.

Once the Ag(220)/Cu_3_P(3360) interface had formed, a structural rearrangement of the phases occurred, which led to the stepwise disappearance of the Ag(111)/Cu_3_P(0330) interface. Our findings show that a potential reason the Ag(220)/Cu_3_P(3360) interface was energetically favored during the rearrangement process can be found in the matching interplanar spacing for planes perpendicular to the addressed heterointerface. Moreover, the simultaneous presence of both heterointerfaces within the same Ag–Cu_3_P nanoparticle led to an inhomogeneous strain distribution within the Ag phase, which might have destabilized the nanoparticle heterostructure and driven the rearrangement process. Furthermore, we observed corner truncation of the faceted Cu_3_P phase over time to reduce the total surface free energy.

We increased the temperature to 500 °C to accelerate the slow rearrangement of the Ag–Cu_3_P nanoparticle at 350 °C and obtained a single Ag(220)/Cu_3_P(3360) interface within the heterostructure after the temperature treatment. The absence of strain fields suggests the presence of a fully coherent heterointerface. The presented results on interface dynamics in Ag–Cu_3_P nanoparticle heterostructures highlight the potential to select specific interfaces in metal–semiconductor heterostructures using templates and varying process parameters. This will help evaluate the impact of their tailored structural and morphological properties on their photocatalytic performance in future experiments.

## Methods

### Ag–Cu Nanoparticle Synthesis and Deposition on MEMS-Based Heating Chips

A home-built spark ablation system with Ag as an anode and Cu as a cathode was used to form bimetallic agglomerates. A N_2_/H_2_ gas flow carried the bimetallic agglomerates from the spark through a furnace for sintering at 850 °C. A differential mobility analyzer system enabled the selection of sintered bimetallic nanoparticles with a diameter of 30 nm for the electrostatic deposition on MEMS-based heating chips. The parameters used for the synthesis and deposition of Ag–Cu nanoparticle heterostructures are in accordance with the parameters used in a recently published study.^55^

### MEMS-Based Heating Chips for *in Situ* TEM Investigations

MEMS-based heating chips supplied by Norcada were used for *in situ* TEM investigations. The chips consist of a thin SiN_*x*_ membrane with an embedded W heating coil for Joule heating. The temperature profile is homogeneous in the central region of the chip, and temperatures up to 1100 °C are achievable. The central region of the chip consists of 19 holes in the SiN_*x*_ membrane with a thin SiN_*x*_ region next to those holes. The Blaze software supplied by Hitachi enables the controlled variation of the temperature in the central region of the chip *via* a constant resistance mode.

### ETEM, TEM Imaging/Movies, and Characterization

The study was performed in a Hitachi HF-3300S ETEM operated at 300 kV. The microscope is equipped with a cold field emission gun and an imaging aberration corrector (CEOS BCOR). A scanning unit enables STEM imaging (annular dark field, bright field, and a secondary electron detector) as well as compositional microanalysis with EDS. The integrated complementary metal–oxide–semiconductor (CMOS) camera (GATAN OneView IS camera) allows for the investigation of dynamic processes by recording HRTEM movies with up to 300 frames/s. An electron dose rate in the range 1000–10 400 e/Å^2^ s was used to acquire TEM images and movies (see Tables S1 and S2). Acquisition parameters used for STEM-EDS elemental maps and high-angle annular dark field (HAADF)-STEM images can be found in Tables S3 and S4. The background pressure next to the sample was ∼1.6·10^–4^ Pa. Furthermore, the nanoparticle heterostructure presented in Figures 1–6 was exposed to a maximum electron dose of ∼1.15·10^8^ e/Å^2^ throughout the whole experiment. Additional information about the ETEM in Lund is available elsewhere.^68^

### Sample Holder and Precursor Supply

Experiments focused on the formation of Ag–Cu_3_P nanoparticles were performed on a custom-built double tilt holder from Hitachi. Mass flow controllers integrated into the gas handling system enabled the controlled supply of PH_3_ directly to the heated area of the MEMS-based heating chip inside the ETEM *via* a side port injector integrated into the microscope column.

### Formation of Ag–Cu_3_P Nanoparticles

The as-deposited Ag–Cu nanoparticles were heated to 500–650 °C, and 2.0 sccm H_2_ (8.9·10^–2^ mmol/min, estimated pressure next to sample: ∼1 Pa) was supplied for ∼5 min *via* the side port injector. The gun valve remained closed during the H_2_ treatment. Subsequently, the temperature was reduced to 350 °C. The H_2_ treatment yielded a high amount of Ag–Cu nanoparticle heterostructures with a single interface and free from surface contamination. In the next step, 0.2 sccm PH_3_ (9·10^–3^ mmol/min, estimated pressure next to sample: ∼6.5·10^–2^ Pa) was supplied to initiate the phase transformation of Cu to Cu_3_P. Finally, the temperature was increased to 500 °C with a heating rate of 5 °C/s to accelerate the growth of the favored heterointerface I5 at the cost of heterointerface I4.

### Multislice Simulations and Exit Wave Function Reconstructions

A stack of 21 HRTEM images in the defocus range from −10 to 10 nm with a defocus step of 1 nm for both the Ag[110]^57^ and Cu_3_P[0001]^58^ projections were simulated. Parameters used for the multislice simulations can be found in Table S5. The images of the simulated image stacks and the acquired focus series (see Table S6) were aligned. Subsequently, for each aligned image stack, an iterative wave function reconstruction was performed. The obtained exit wave functions were used to refine the alignment of the corresponding image stack. In total, three refinement–reconstruction cycles were performed for each image stack. The obtained exit wave functions were slightly adjusted *via* the integrated aberration corrector. Parameters used for the exit wave function reconstructions can be found in Table S7.

### Geometrical Phase Analysis

The phase image of the reconstructed exit wave function was used to perform a GPA. The Cu_3_P(1120) reflection was selected as the smallest g-vector in the power spectrum. The Ag(111) and Ag(111) reflections were chosen as g-vectors to prepare the strain map. Refinement was performed in the central region of the Ag phase. The axes were rotated in such a way that the *x*-axis is equal to the Ag[220] direction (vector addition of both chosen g-vectors). The strain map visualizes strain in the Ag[220] direction, and the strain limits were set to ±0.08.

### Simulation of Electron Diffraction Patterns

For each power spectrum, the associated scale bar was used to determine the camera length for the simulation of the electron diffraction pattern. Further parameters used for the simulation of electron diffraction patterns can be found in Table S8. The expansion of the lattice at different temperatures has been considered for the electron diffraction pattern simulations of cubic Ag and Cu by using thermal expansion coefficients found in the literature.^57^ In contrast to the Ag and Cu phases, the thermal expansion of Cu_3_P upon heating was not considered for the simulation of the associated electron diffraction pattern due to the missing thermal expansion coefficient of hexagonal Cu_3_P in the literature.

### Data Processing/Software

TEM images and movies (In-situ player) were acquired and processed with DigitalMicrograph from Gatan (Version 3.43.3213.0). For the HRTEM images extracted from Movie S1, the specific frames associated with selected times were averaged with the same amount of frames straight before and after the event (making a total of 11 (Figures 2 and 4)/3 (Figure 3) consecutive frames for averaging). Power spectra obtained from DigitalMicrograph were overlaid with simulated electron diffraction patterns using SingleCrystal from CrystalMaker Software Ltd. (Version 4.1.0). Atomic models were built with CrystalMaker from CrystalMaker Software Ltd. (Version 4.1.0). For acquiring and processing HAADF-STEM and EDS data, AZtec from Oxford Instruments Nanotechnology Tools Ltd. (Version 3.3) was used. Multislice simulations were performed with jems from P. Stadelmann (Version 4.9131U2020b31). Strain++ (Version 1.6), maintained by J. J. P. Peters, was used to prepare strain maps. In this software, strain is measured using a GPA algorithm described in the literature.^64^ The exit wave function reconstructions were performed with IWFR for DigitalMicrograph from HREM Research Inc. (Version 2.0). Figures for this publication were prepared using Adobe Photoshop from Adobe (Version 22.1.0). Movies in the Supporting Information were compressed using ImageJ (Version 1.52a).

## Supporting Information Available

Figures of HRTEM images, STEM-EDS elemental maps, power spectra, EDS sum spectra, HAADF-STEM image, HRTEM simulations for the Cu_3_P phase, comparison of experimental data with simulations for the Cu_3_P phase, HRTEM simulations for the Ag phase, comparison of experimental data with simulations for the Ag phase, confirmation of the comparisons’ validity using the squared moduli of the Fourier transform of the exit wave function and simulated SAED patterns for the estimated tilts around specific crystal directions, and atomic models of the Ag(111)/Cu_3_P(0330) interface and tables of (acquisition) parameters used for HRTEM images and movies, STEM-EDS elemental maps, HAADF-STEM images, multislice simulations, the acquired focus series, exit wave function reconstructions, and simulations of electron diffraction patterns (PDF)
Movie of Cu–Cu_3_P phase transformation corresponding to Figures 2–4 (AVI)
Movie of replacement of heterointerface I4 by heterointerface I5 during temperature increase to 500 °C (AVI)

## Supplementary Material

ja1c09179_si_001.pdf

ja1c09179_si_002.avi

ja1c09179_si_003.avi

## Abbreviations Used

TMP: transition metal phosphide
LSPR: localized surface plasmon resonance
HER: hydrogen evolution reaction
TEM: transmission electron microscopy
ETEM: environmental transmission electron microscope
MOCVD: metal–organic chemical vapor deposition
HRTEM: high-resolution transmission electron microscopy
EDS: energy dispersive X-ray spectroscopy
MEMS: microelectromechanical systems
STEM: scanning transmission electron microscopy
GPA: geometrical phase analysis
SAED: selected area electron diffraction
CMOS: complementary metal–oxide–semiconductor
HAADF: high-angle annular dark field
