# Supplementary material for: Interface Dynamics in Ag–Cu3P Nanoparticle Heterostructures
Source: J Am Chem Soc. 2021 Dec 24;144(1):248–58. doi: 10.1021/jacs.1c09179 (PMC8759066; doi:10.1021/jacs.1c09179)
Supplement: Supplementary file 1 — ja1c09179_si_001.pdf [file ja1c09179_si_001.pdf]

Supporting Information for

Interface Dynamics in Ag-Cu_3P Nanoparticle Heterostructures

Michael S. Seifner,*,†,‡ Markus Snellman,‡,§ Ofentse A. Makgae,†,‡ Krishna Kumar,†,‡ Daniel Jacobsson,†,‡,∥ Martin Ek,†,‡ Knut Deppert,‡,§ Maria E. Messing,‡,§ and Kimberly A. Dick*,†,‡

† Centre for Analysis and Synthesis, Lund University, Box 124, 22100 Lund, Sweden

‡ NanoLund, Lund University, Box 118, 22100 Lund, Sweden

§ Solid State Physics, Lund University, Box 118, 22100 Lund, Sweden

∥ National Center for High Resolution Electron Microscopy, Lund University, Box 124, 22100 Lund, Sweden

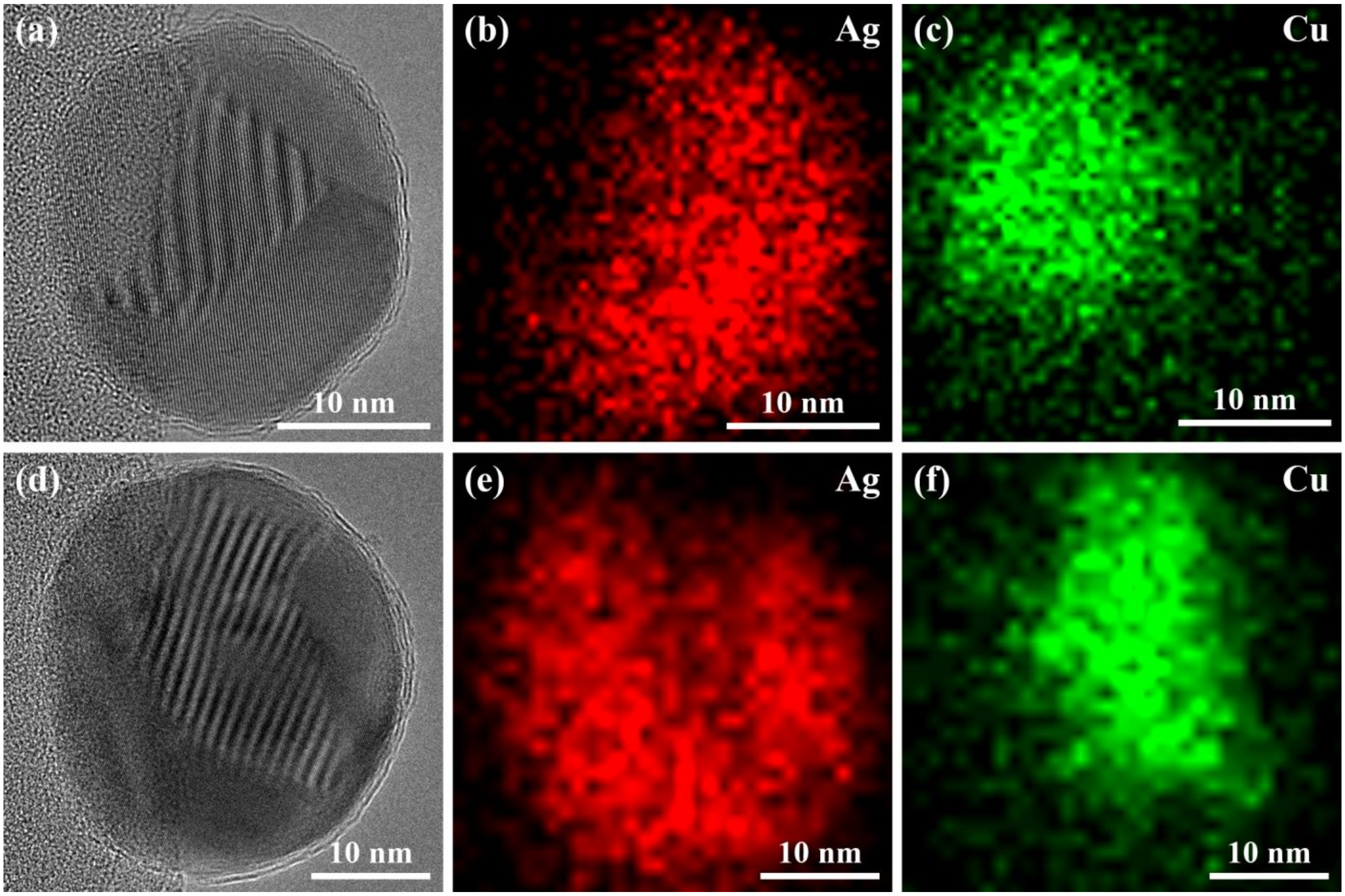

Figure S1. (a+d) HRTEM images of as-deposited bimetallic Ag-Cu nanoparticles heated up to 350 °C. Multiple heterointerfaces are present in each nanoparticle, and oxides/C-based contamination cover(s) the surfaces. The diameters of both nanoparticles are in good agreement with the chosen diameter of 30 nm for the deposition of bimetallic nanoparticles generated in a spark ablation system on the MEMS-based heating chip. STEM-EDS elemental maps of (b+e) Ag ($L\alpha_1$) and (c+f) Cu ($K\alpha_1$) corresponding to the nanoparticles presented in (a+d). 8 Ag-Cu nanoparticles (including those shown here) were analyzed to determine an average composition of 56.24±9.51 atom % Ag (L series) and 43.76±9.51 atom % Cu (K series).

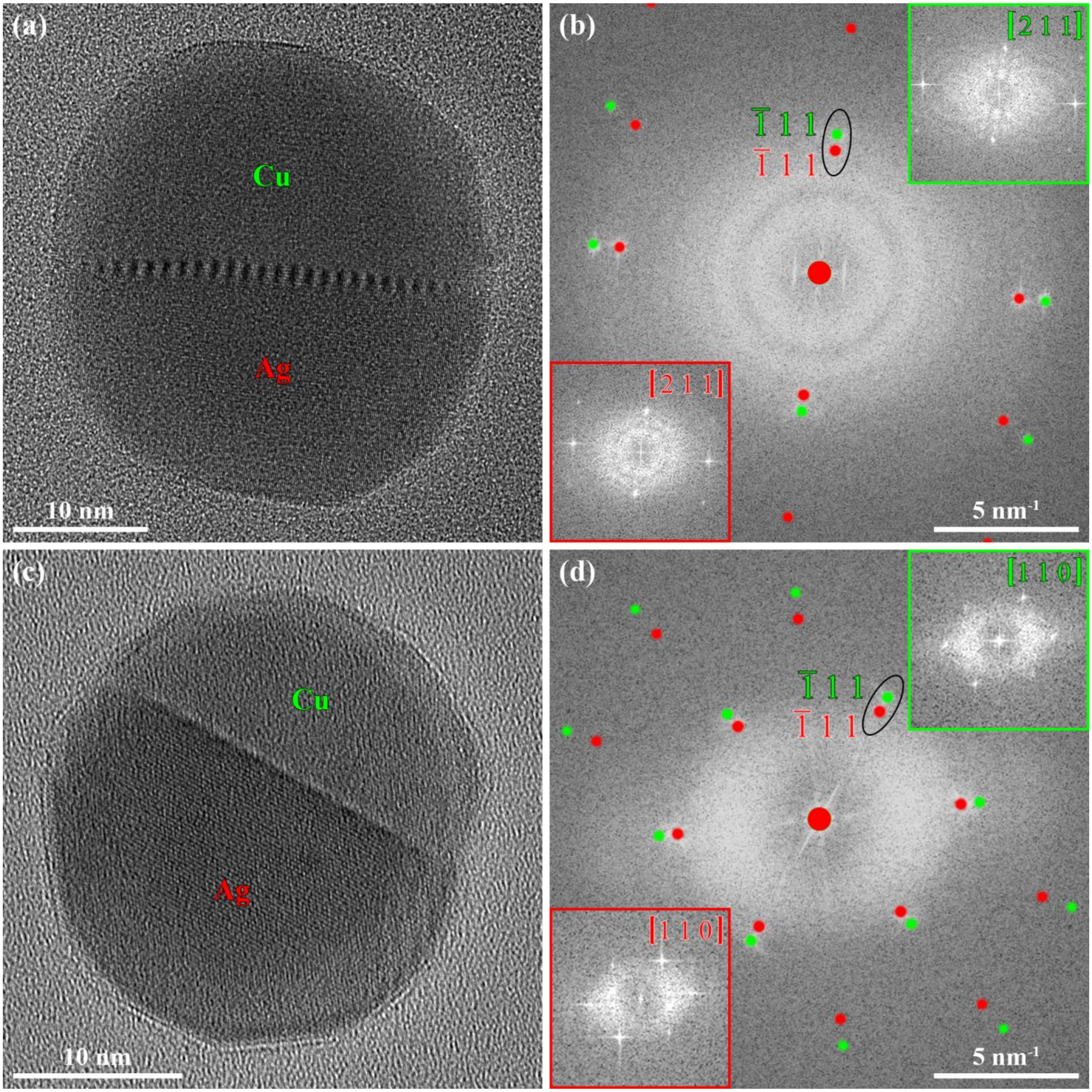

Figure S2. (a+c) HRTEM images of Ag-Cu nanoparticle heterostructures observed after H_2 treatment at 500-650 °C. Both heterointerfaces are tilted close to parallel to the electron beam. (b+d) All phases are oriented close to one of their zone axes as highlighted in the insets, and the corresponding power spectra with overlaid simulated electron diffraction patterns of the cubic Ag (red) and Cu (green) phases reveal the involvement of {111} facets in the heterointerface formation.

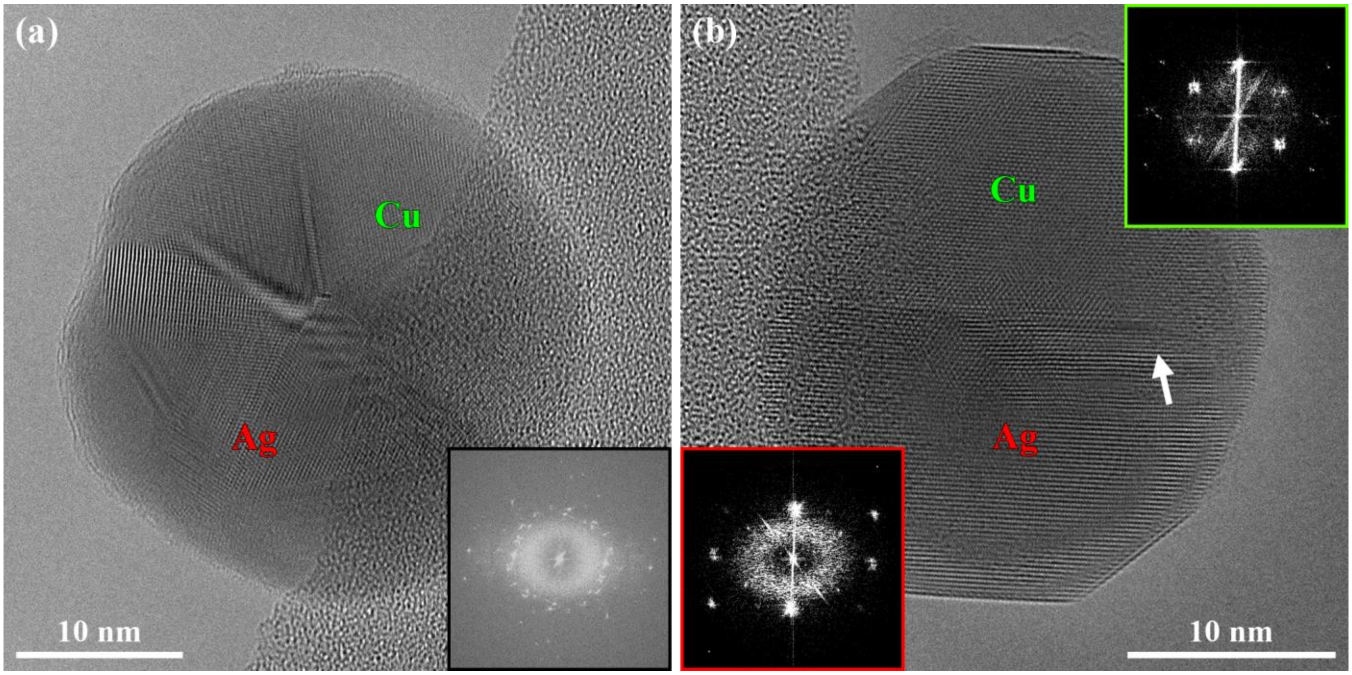

Figure S3. HRTEM images of Ag-Cu nanoparticles without a sharp Ag{111}/Cu{111} interface after H_2 treatment at 500-650 °C. (a) Both phases reveal a high number of defects, which is supported by the corresponding power spectrum as inset. (b) The presence of crystal defects usually leads to rough heterointerfaces highlighted by a white arrow. The power spectra of the Ag (red, bottom left) and Cu (green, top right) phases shown as insets of (b) are altered in brightness and contrast to reveal the addressed presence of defects.

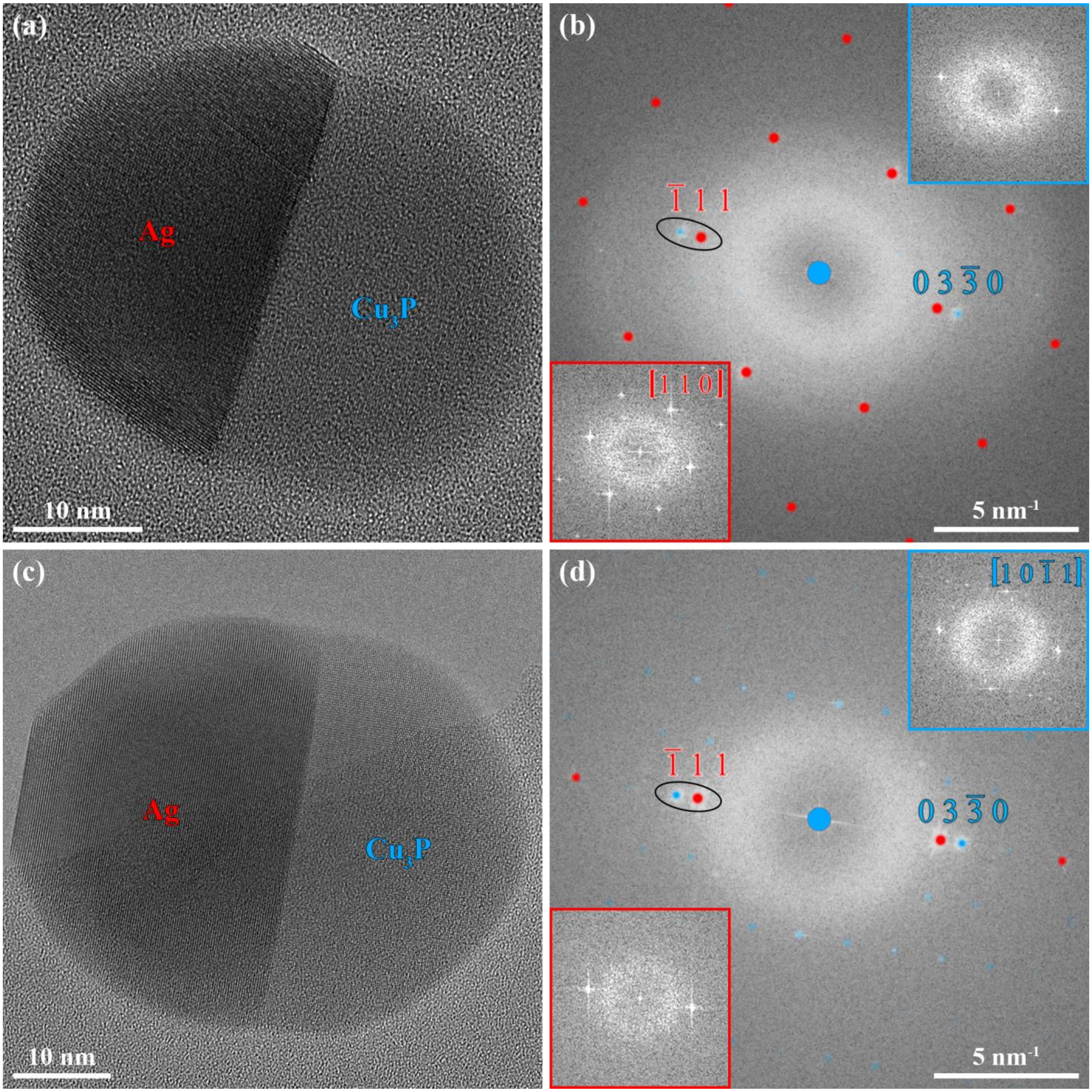

Figure S4. (a) HRTEM image of a Ag-Cu_3P nanoparticle heterostructure with a single interface. (b) The power spectrum corresponding to (a) with overlaid simulated electron diffraction patterns confirms the presence of a Ag($\bar{1}11$)/Cu_3P($0\bar{3}30$) interface. The insets in (b) reveal the Ag phase (red, bottom left) tilted close to its [110] zone axis and the Cu_3P phase (blue, top right) tilted off its zone axis. (c) HRTEM image of a Ag-Cu_3P nanoparticle heterostructure with a significantly larger diameter of ~45 nm. (d) The heterointerface is again formed by Ag($\bar{1}11$) and Cu_3P($0\bar{3}30$) planes as highlighted by the power spectrum associated with (c) with overlaid simulated electron diffraction patterns. The insets in (d) show that the Cu_3P phase (blue, top right) is tilted close to its [$10\bar{1}1$] zone axis and the Ag phase (red, bottom left) is tilted off its zone axis.

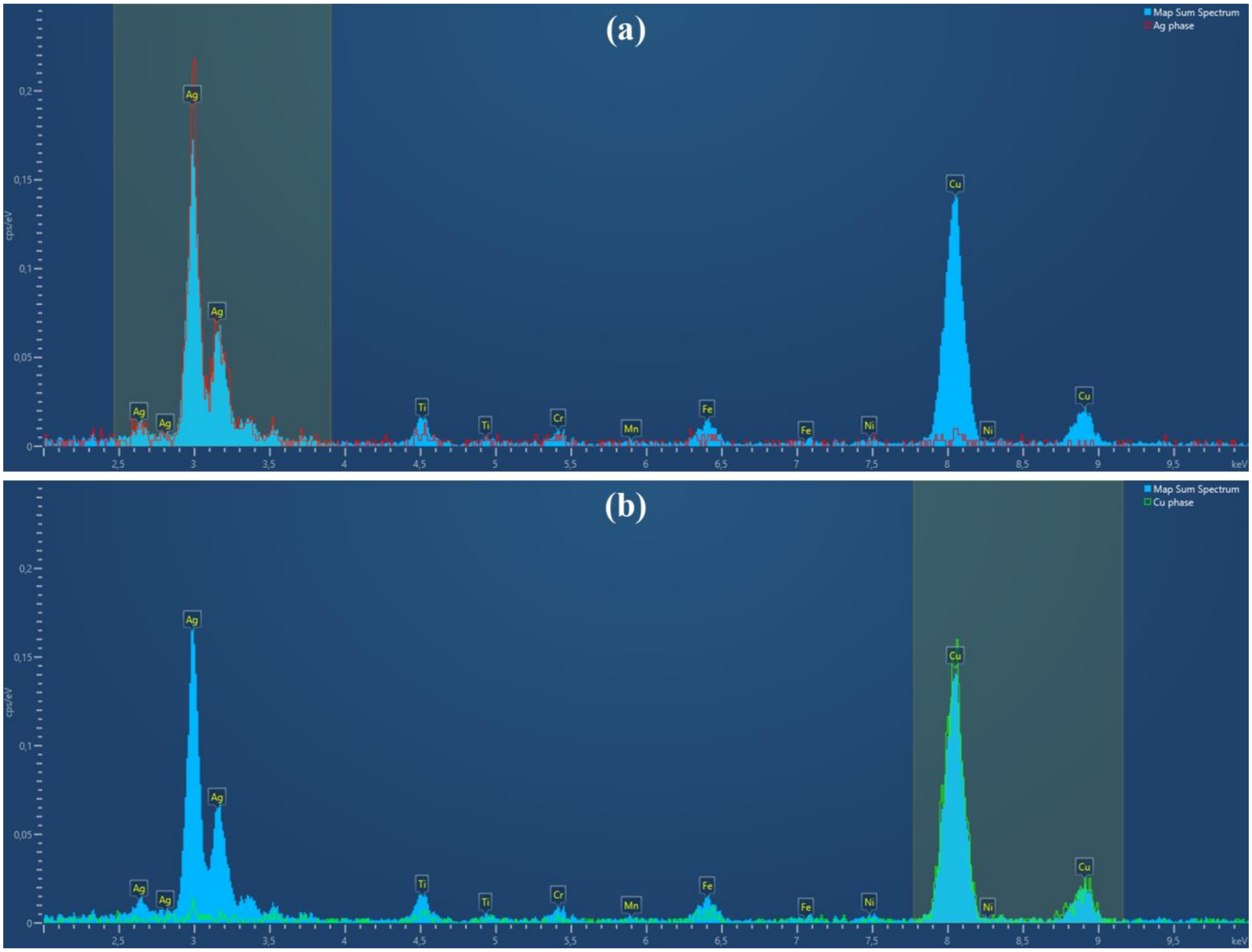

Figure S5. EDS sum spectra of the STEM-EDS measurement of the Ag-Cu nanoparticle presented in Figure 1 of the main manuscript. The EDS sum spectra of selected areas within the (a) Ag and (b) Cu phases are overlaid with the overall sum spectrum. The yellow areas represent the energy ranges used for normalization. Both elements show a very low solubility in each other (~3 atom %), which is in good agreement with the Ag-Cu binary phase diagram[1]. The additional EDS peaks (Ti, Cr, Mn, Fe, and Ni) obtained due to electron scattering can be allocated to the side port injector and the TEM holder material.

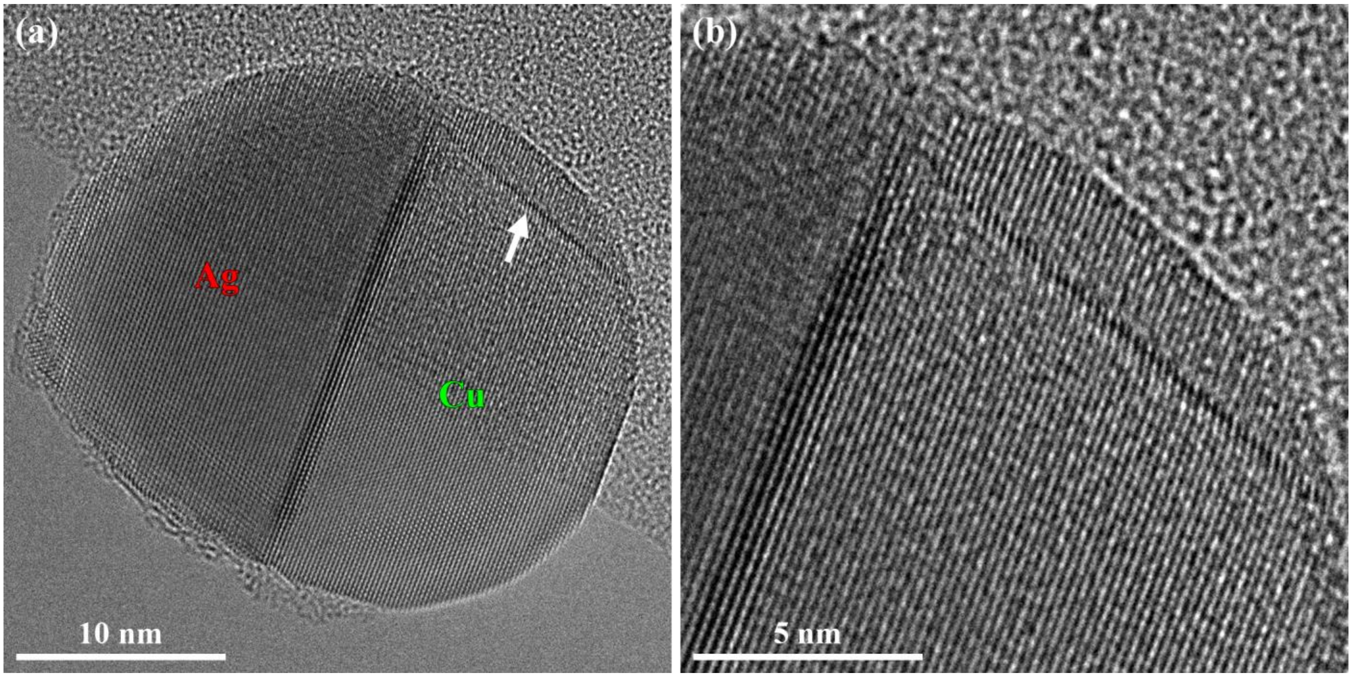

Figure S6. (a) HRTEM image of the same Ag-Cu nanoparticle as shown in Figure 1 of the main manuscript acquired with underfocus. The white arrow highlights the presence of a defect in the region where heterointerface I5 formed after the complete transformation of Cu to Cu_3P at a later stage of the experiment. (b) A zoomed-in region of the HRTEM image in (a) for a better visualization of the addressed defect.

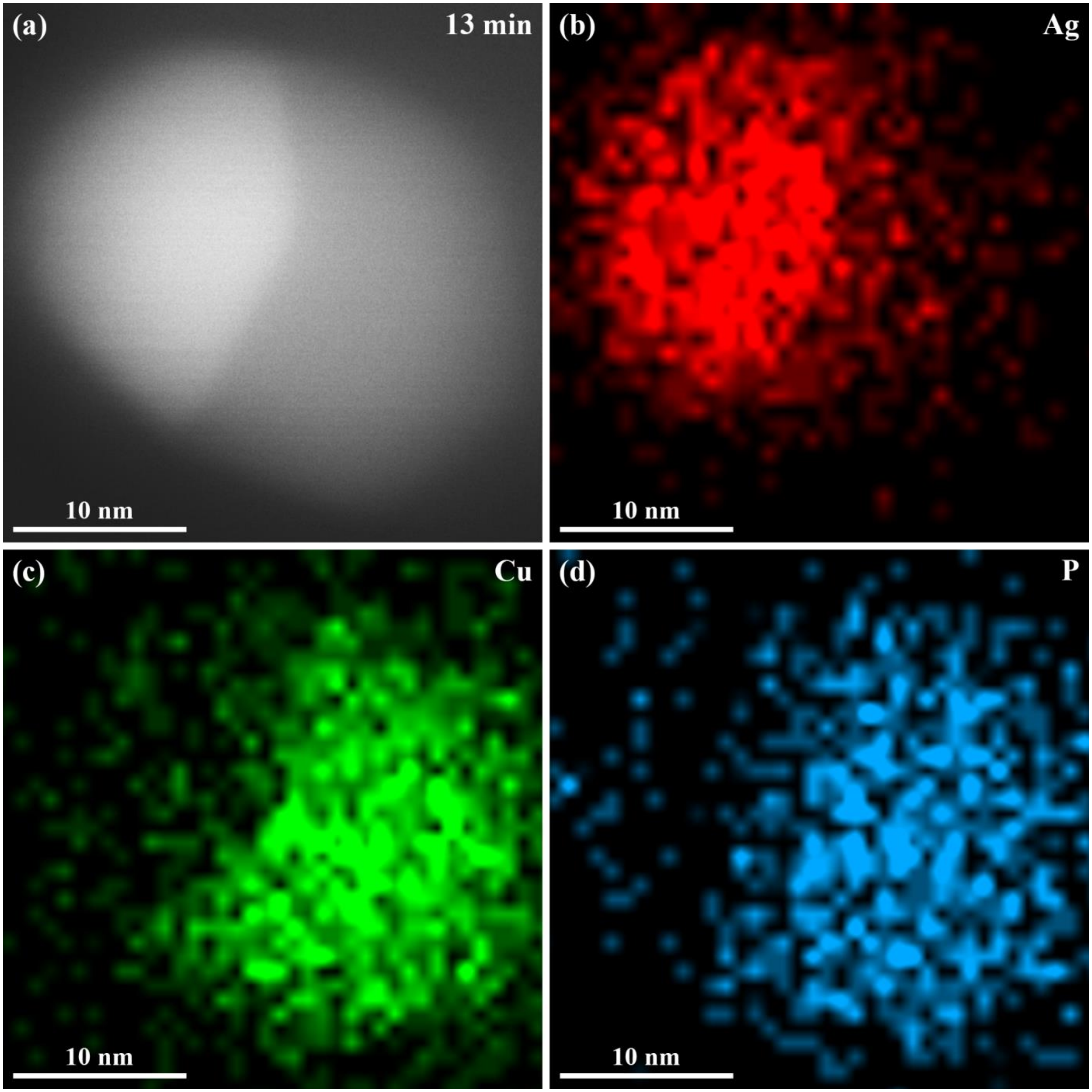

Figure S7. (a) HAADF-STEM image of the Ag-Cu_3P nanoparticle heterostructure presented in Figure 4a of the main manuscript and acquired 13 min after starting the PH_3 supply. STEM-EDS elemental maps of (b) Ag ($L\alpha_1$), (c) Cu ($K\alpha_1$) and (d) P ($K\alpha_1$) confirm the exclusive chemical reaction of PH_3 with Cu. The Ag-Cu_3P nanoparticle heterostructure contains 38.82 atom % Ag (L series), 41.82 atom % Cu (K series) and 19.36 atom % P (K series). The Cu:P atomic ratio is significantly lower than 3:1 (2.16:1), which is in agreement with previous studies suggesting a high concentration of Cu vacancies in hexagonal Cu_3P[2-3]. The Cu_3P phases in 8 Ag-Cu_3P nanoparticle heterostructures (including the one presented here) synthesized under similar conditions have been analyzed to determine an average composition of 71.71±2.52 atom % Cu (K series) and 28.29±2.52 atom % P (K series) giving an average Cu:P atomic ratio of 2.53:1.

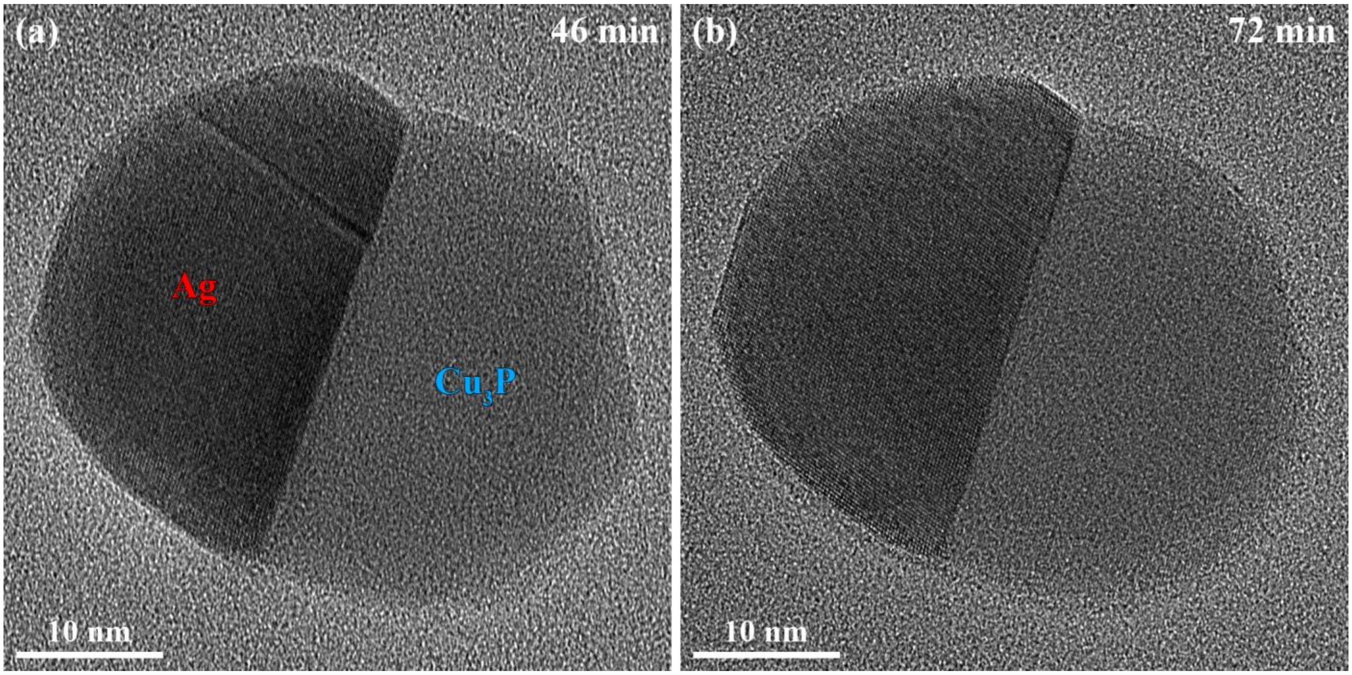

Figure S8. HRTEM images of the same Ag-Cu_3P nanoparticle heterostructure as shown in Figure S4a acquired (a) 46 min and (b) 72 min after stopping the supply of PH_3 at 350 °C. (a) Faceting of the Cu_3P phase (right) of the nanoparticle heterostructure is visible. (b) Corner truncation leads to a physical transformation of the Cu_3P phase.

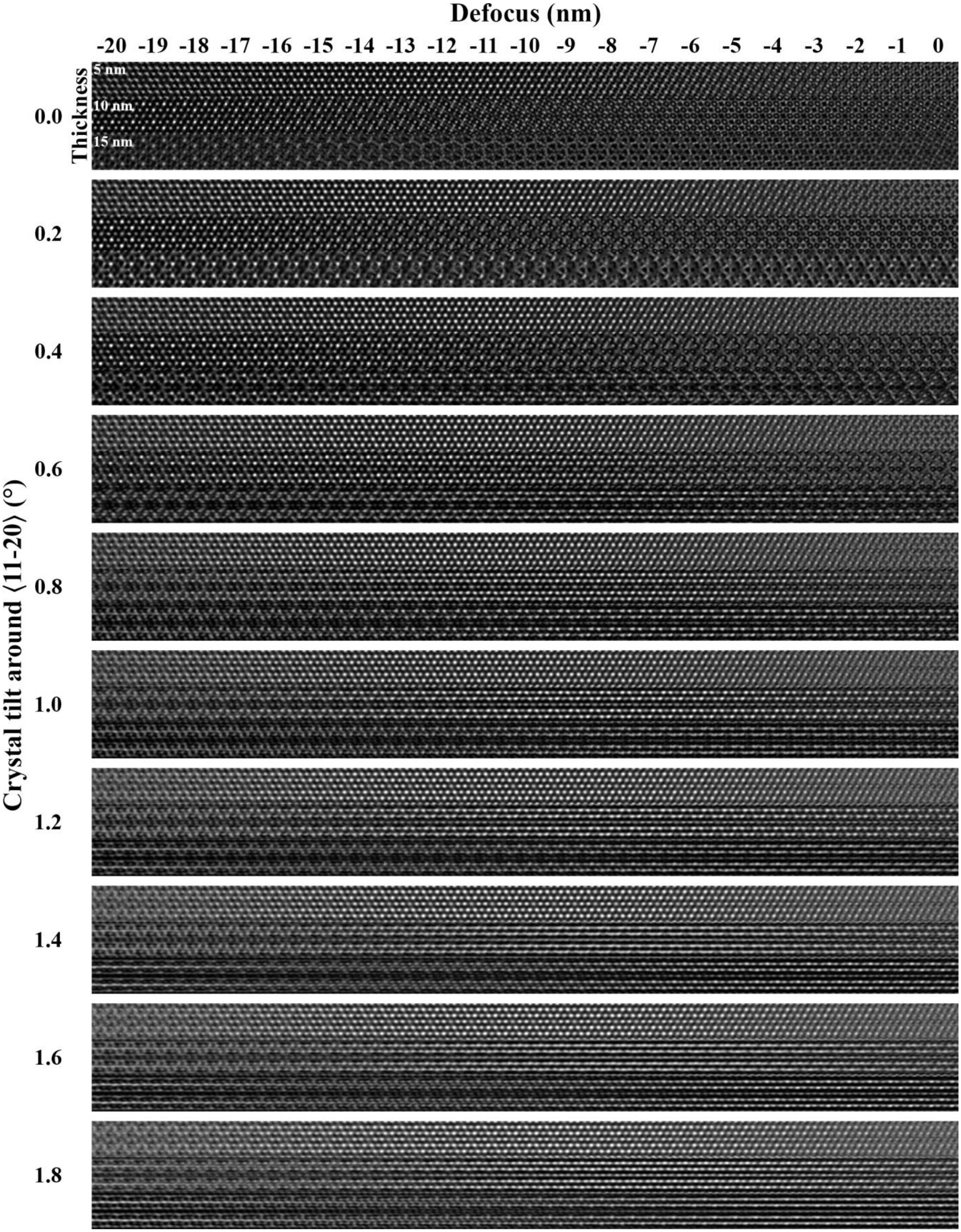

Figure S9. Simulation of HRTEM images of a Cu_3P crystal oriented in its [0001] zone axis with different thicknesses for specific defocus values and crystal tilts around the ⟨11$\bar{2}$0⟩ direction.

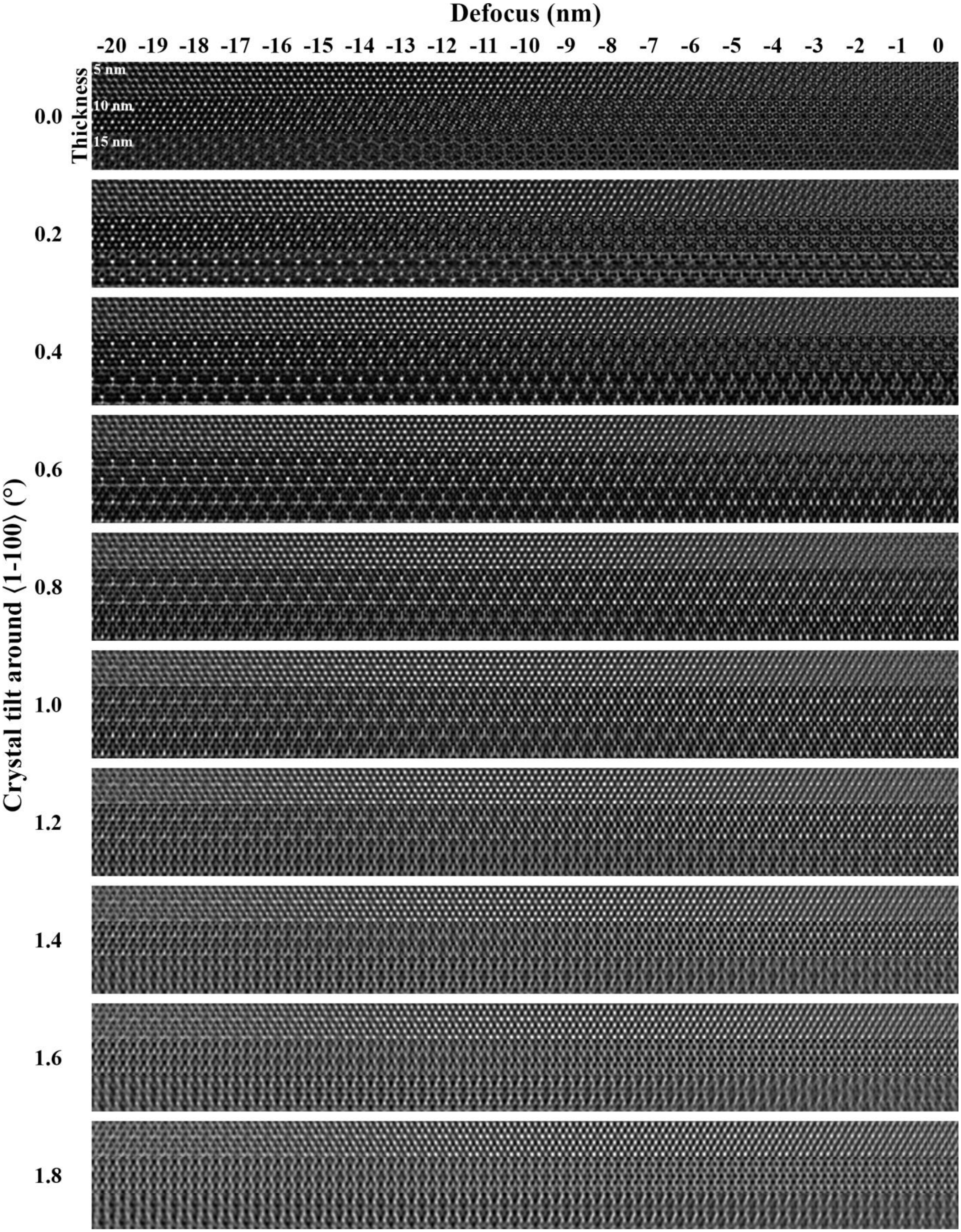

Figure S10. Simulation of HRTEM images of a Cu_3P crystal oriented in its [0001] zone axis with different thicknesses for specific defocus values and crystal tilts around the ⟨1$\bar{1}$00⟩ direction.

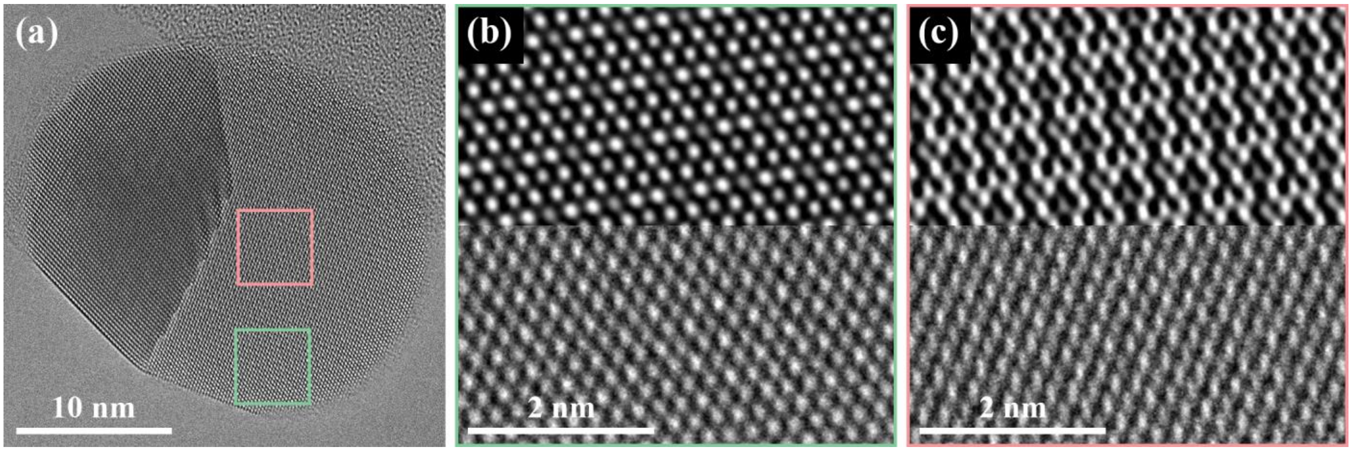

Figure S11. (a) A selected HRTEM image of the acquired focus series (see Table S6) with a defocus value of -4.66 nm (-5.10 nm according to the refinement of the exit wavefunction reconstruction (see Table S7)). The (b) thin and (c) thick regions of the Cu_3P crystal are indicated by green and pink rectangles in (a). The upper halves of the HRTEM images in (b) and (c) are matching simulations. The matching simulations (defocus: -12 nm, 1.6° tilt around the Cu_3P [$1\bar{1}00$] direction (see Figure 5)) suggest the presence of (b) 5 nm and (c) 10 nm thick regions in the Cu_3P crystal. A possible explanation for the different defocus values of the acquired image and the matching simulations could be a slight variation of the height along the nanoparticle heterostructure. The same parameters chosen for the multislice simulations to reconstruct the exit wavefunction are used for the HRTEM simulations (see Table S5). Straight before acquiring the focus series, the aberrations have been measured and are used for the HRTEM simulations (A_1: 3.557 nm/-177.9°, B_2: 22.13 nm/97.1°, A_2: 52.29 nm/39.6°, C_S: 16.47 µm).

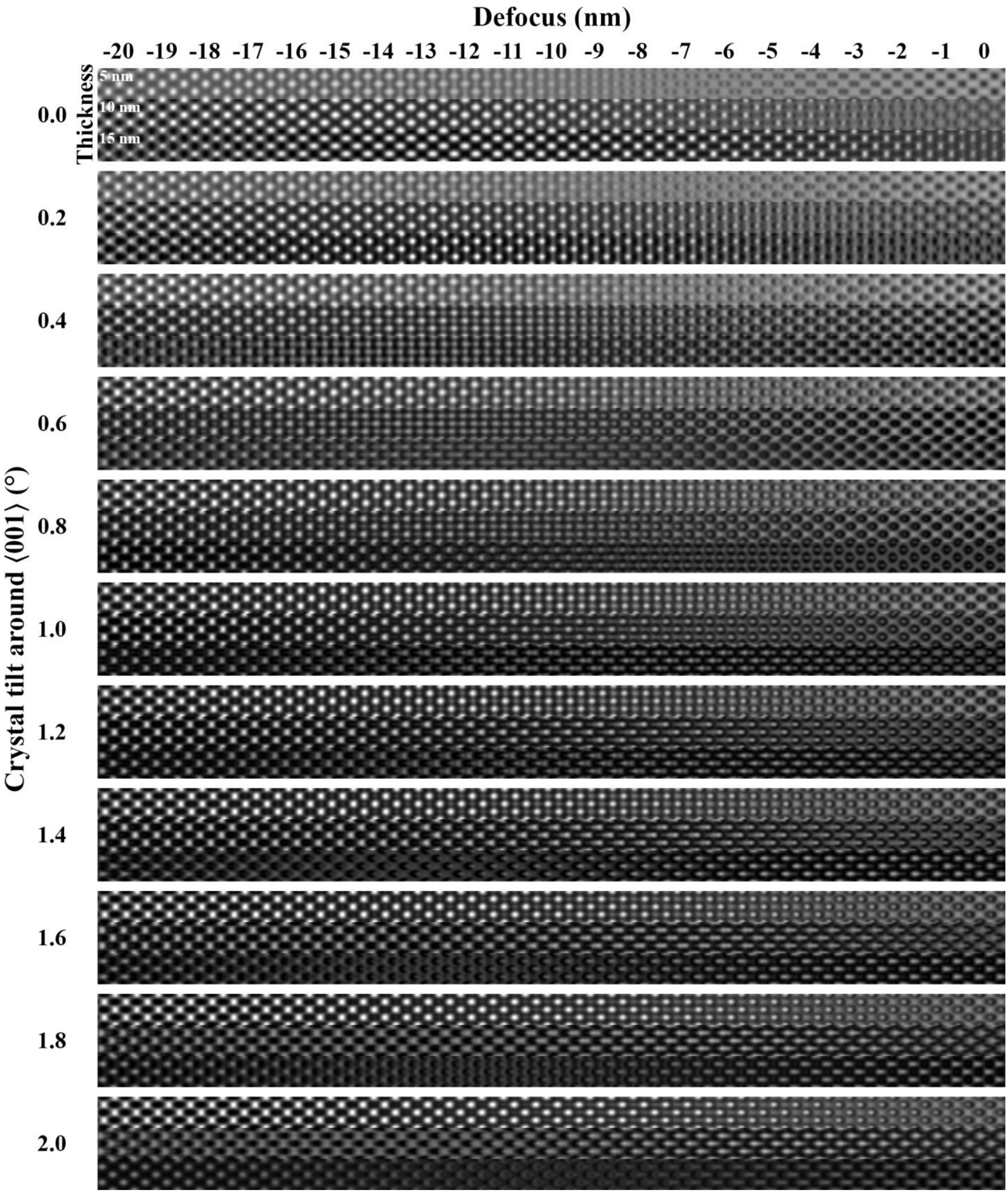

Figure S12. Simulation of HRTEM images of a Ag crystal oriented in its [110] zone axis with different thicknesses for specific defocus values and crystal tilts around the ⟨001⟩ direction.

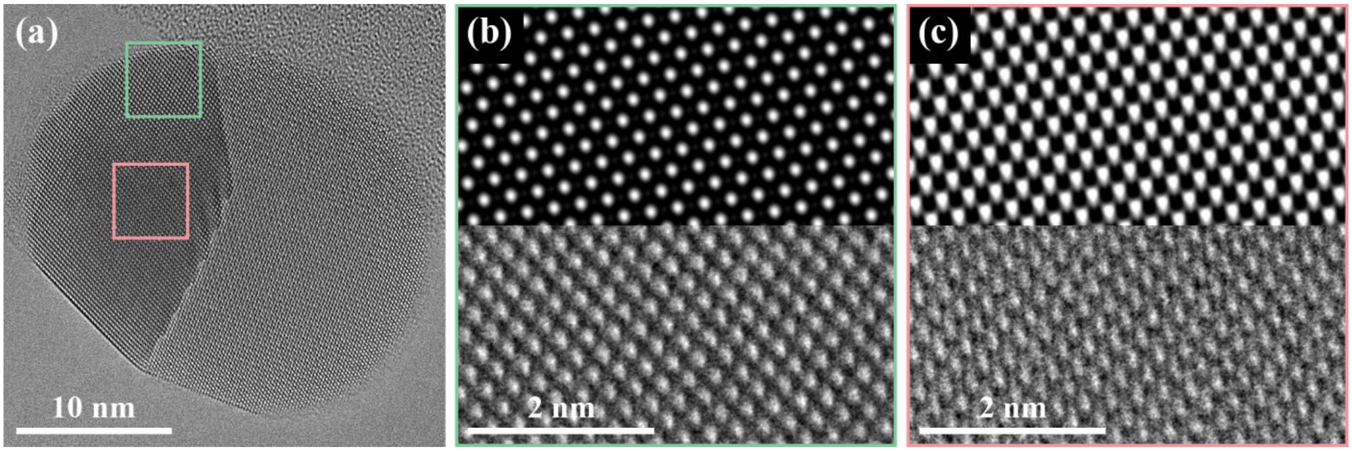

Figure S13. (a) HRTEM image of the acquired focus series (see Table S6) with a defocus value of -4.66 nm (-5.10 nm according to the refinement of the exit wavefunction reconstruction (see Table S7)). The (b) thin and (c) thick regions of the Ag crystal are indicated by green and pink rectangles in (a). The upper halves of the HRTEM images in (b) and (c) are simulations for (b) 5 nm and (c) 10 nm with the same defocus values (-12 nm) and extents of tilt (1.6°) around the Ag [001] direction (see Figure 5) as determined in Figure S11 to match the Cu_3P phase. Details about the multislice simulations are presented in Figure S11.

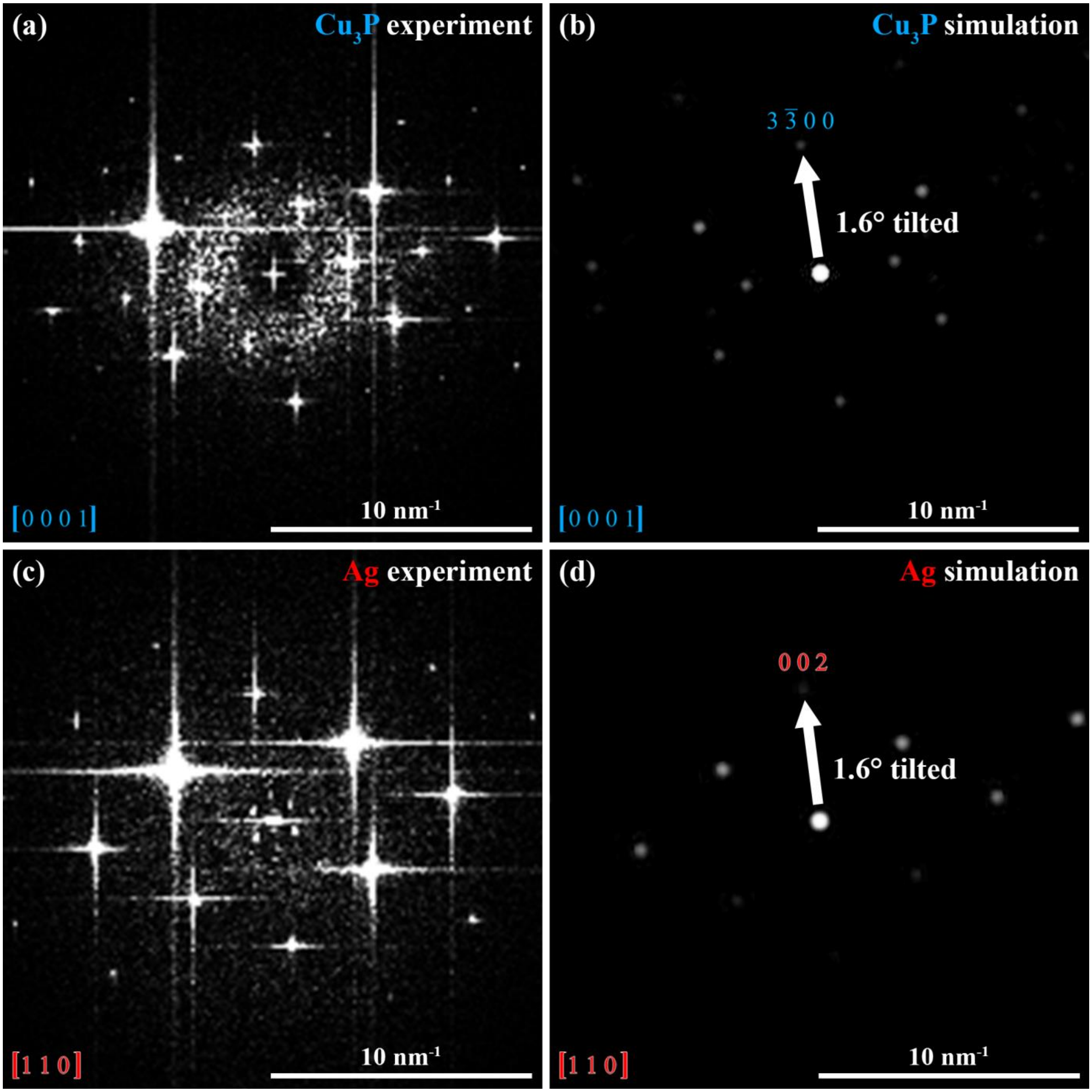

Figure S14. The squared moduli of the Fourier transform of the exit wavefunction for the (a) Cu_3P and (c) Ag phases are compared with simulated SAED patterns (*jems*, multislice simulations) for the (b) Cu_3P and (d) Ag phases with the determined tilt values of 1.6° around the (b) Cu_3P [1$\bar{1}$00] and (d) Ag [001] directions to confirm the results presented in Figures S9-13. The comparisons lead to the conclusion that both phases are slightly tilted off their zone axes in the same direction and to the same extent (not more than 1.6°). If the investigated areas of the Ag and Cu_3P crystals were slightly thicker than expected (>10 nm), the extent of tilting off their zone axes would even be less. Therefore, the planes forming heterointerface I5 are expected to be very close to parallel. Simulations have been performed for ~15 nm thick crystals. For better qualitative comparisons of the experimental data with the simulations, the brightness and contrast of the simulated SAED patterns have been slightly modified.

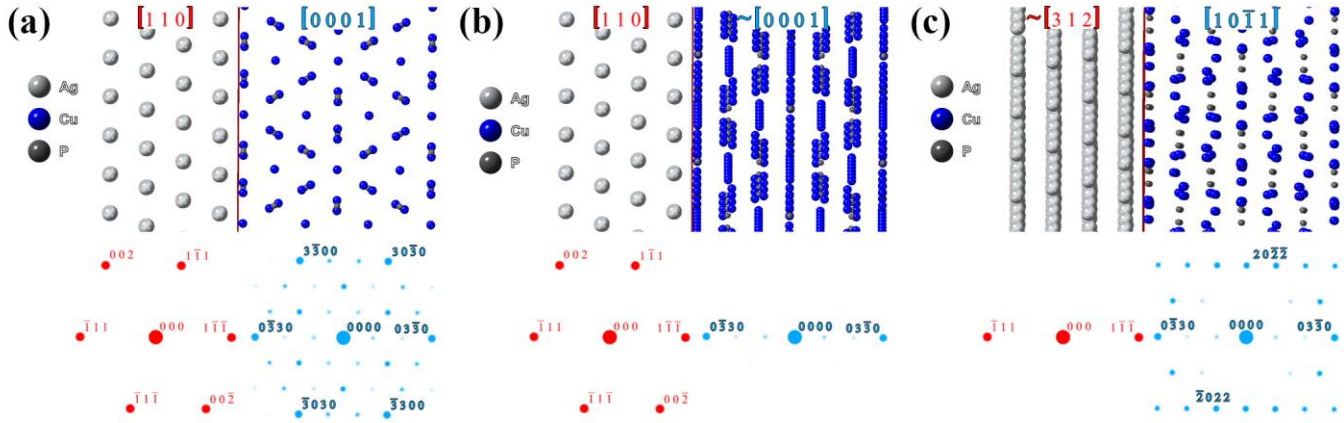

Figure S15. Atomic models of the $Ag(\bar{1}11)/Cu_3P(0\bar{3}30)$ interface and corresponding simulated electron diffraction patterns for nanoparticle heterostructures shown in (a) Figure 5a, (b) Figure S4a, and (c) Figure S4c. (a) The observed in-plane angular mismatch is not considered for this atomic model. The Ag phase is oriented in its [110] zone axis, while the Cu_3P phase is oriented in its [0001] zone axis. Note that the phases are limited in their capability to rotate against each other due to the presence of heterointerface I5. In contrast to (a), the Ag-Cu_3P nanoparticle heterostructures in (b+c) have a single $Ag(\bar{1}11)/Cu_3P(0\bar{3}30)$ interface. (b) The Ag phase is again oriented in its [110] zone axis, while the Cu_3P phase is slightly rotated against the Ag phase (~3.5° rotation of the Cu_3P phase in (a) to match the power spectrum in Figure 4b). Therefore, we conclude that the phases' freedom to rotate against each other yields a slightly different arrangement than observed for heterointerface I4 in Figure 5a. (c) A 48°-rotation of the model in (b) matches the experimental observations in Figures S4c and S4d. Consequently, there likely exists a preferred arrangement for $Ag(\bar{1}11)$ and $Cu_3P(0\bar{3}30)$ planes forming a heterointerface.

Table S1. Acquisition parameters used for HRTEM images.

HRTEM image	Electron dose rate (e/Å^2 s)
Figure 1a	~3500
Figure 5a	~3500
Figure 6a	~4600
Figure S1a	~2100
Figure S1d	~1000
Figure S2a	~10400
Figure S2c	~5100
Figure S3a	~9800
Figure S3b	~7300
Figure S4a	~4300
Figure S4c	~2700
Figure S6a	~3500
Figure S8a	~4100
Figure S8b	~4200

Table S2. Acquisition parameters used for HRTEM movies.

HRTEM movie	Electron dose rate (e/Å^2 s)	Frame rate (frames/s)
Movie S1	~7800	20
Movie S2	~1600	20

Table S3. Acquisition parameters used for STEM-EDS elemental maps.

STEM-EDS elemental map	Pixel size (nm)	Binning	Time (s)	Total counts	Scans
Figures 1c+d	0.179	4	196	128566	11
Figures S1b+c	0.139	4	185	131196	7
Figures S1e+f	0.139	8	130	102533	3
Figures S7b-d	0.208	4	227	139283	17

Table S4. Acquisition parameters used for HAADF-STEM images.

HAADF-STEM image	Pixel size (nm)	Dwell time (μs)	Scans
Figure S7a	0.0521	10	1

Table S5. Parameters used for multislice simulations.

Accelerating voltage	300 kV
Coefficient of chromatic aberration, C_C	1.500 mm
Coefficient of spherical aberration, C_S	0.016 mm
Energy spread	0.600 eV
Camera pixel size	15 μm
Camera rows/columns	4096/4096
Magnification	1500000
Illumination model	Envelope

Beam half convergence	1.0 mrad
Defocus spread	2.0 nm
Defocus minimum	-10 nm
Defocus step	1.0 nm
Defocus number	21
Noise	0 %
Multislice iterations: start after	0
Multislice iterations: number	1
Multislice iterations: increment	5 (Ag)/4 (Cu_3P)
Specimen thickness	2.88 nm (Ag)/2.85 nm (Cu_3P)
Atomic form factor	WKc.
Potential generation	Direct
Frozen lattice	Bicubic
Temperature	273.15 K
Aperture diameter	40.0 nm^{-1}

Table S6. Acquisition parameters used for the focus series.

Electron dose rate ($e/Å^2$ s)	Exposure time (s)	Defocus minimum (nm)	Defocus step (nm)	Defocus number
~3600	1.0	-9.32	4.66	11

Table S7. Parameters used for exit wavefunction reconstructions.

Accelerating voltage	300 kV
Start defocus	-10 nm (simulations)/-9.32 nm (experiment)
Defocus step	1 nm (simulations)/4.66 nm (experiment)
Total images	21 (simulations)/11 (experiment)
Refinement-reconstruction cycles	3
Maximum iteration cycles	10
Noise control	0.5
Coefficient of spherical aberration, C_S	0.016 mm (simulations)/0.01647 mm (experiment)
Defocus spread	2.0 nm
Beam convergence	1.0 mrad

Table S8. Parameters used for simulations of electron diffraction patterns.

Instrument: voltage	300 kV
Instrument: convergence	1°
Detector: spot size	0.02 $Å^{-1}$
Detector: saturation	100
Detector: gamma	2
Sample: thickness	100 Å
Sample: vol. fraction	100.0 %

References

1. Subramanian, P. R.; Perepezko, J. H., The Ag-Cu (Silver-Copper) System. *J. Phase Equilib.* **1993,** *14* (1), 62-75.
2. De Trizio, L.; Gaspari, R.; Bertoni, G.; Kriegel, I.; Moretti, L.; Scotognella, F.; Maserati, L.; Zhang, Y.; Messina, G. C.; Prato, M.; Marras, S.; Cavalli, A.; Manna, L., $Cu_{3-x}P$ Nanocrystals as a Material Platform for Near-Infrared Plasmonics and Cation Exchange Reactions. *Chem. Mater.* **2015,** *27* (3), 1120-1128.
3. Wolff, A.; Doert, T.; Hunger, J.; Kaiser, M.; Pallmann, J.; Reinhold, R.; Yogendra, S.; Giebeler, L.; Sichelschmidt, J.; Schnelle, W.; Whiteside, R.; Gunaratne, H. Q. N.; Nockemann, P.; Weigand, J. J.; Brunner, E.; Ruck, M., Low-Temperature Tailoring of Copper-Deficient $Cu_{3-x}P$—Electric Properties, Phase Transitions, and Performance in Lithium-Ion Batteries. *Chem. Mater.* **2018,** *30* (20), 7111-7123.
